# Probing amplified Josephson plasmons in YBa_2_Cu_3_O_6+x_ by multidimensional spectroscopy

**DOI:** 10.1038/s41535-025-00776-1

**Published:** 2025-06-06

**Authors:** N. Taherian, M. Först, A. Liu, M. Fechner, D. Pavicevic, A. von Hoegen, E. Rowe, Y. Liu, S. Nakata, B. Keimer, E. Demler, M. H. Michael, A. Cavalleri

**Affiliations:** 1 Max Planck Institute for the Structure and Dynamics of Matter, 22761 Hamburg, Germany; 2 Max Planck Institute for Solid State Research, 70569 Stuttgart, Germany; 3 Institute for Theoretical Physics, ETH Zurich, 8092 Zurich, Switzerland; 4 Department of Physics, University of Oxford, Oxford, OX1 3PU UK

**Keywords:** Superconducting properties and materials, Superconducting properties and materials

## Abstract

The nonlinear driving of collective modes in quantum materials can lead to a number of striking non-equilibrium functional responses, which merit a comprehensive exploration of underlying dynamics. However, the coherent coupling between nonlinearly-driven modes frequently involves multiple mode coordinates at once, and is often difficult to capture by one-dimensional pump probe spectroscopy. One example is phonon-mediated amplification of Josephson plasmons in YBa_2_Cu_3_O_6+x_, a phenomenon likely associated with the mysterious superconducting-like optical response observed in this material. Here, we report two-dimensional nonlinear spectroscopy measurements in driven YBa_2_Cu_3_O_6+x_. We excite apical oxygen phonons with *pairs* of mutually-delayed carrier envelope phase stable mid-infrared pump pulses, and detect time-modulated second-order nonlinear optical susceptibility. We find that the driven phonons parametrically amplify coherent pairs of fluctuating opposite-momentum Josephson plasma polaritons, corresponding to a squeezed state of the Josephson plasma.

## Introduction

**Fig. 1:**
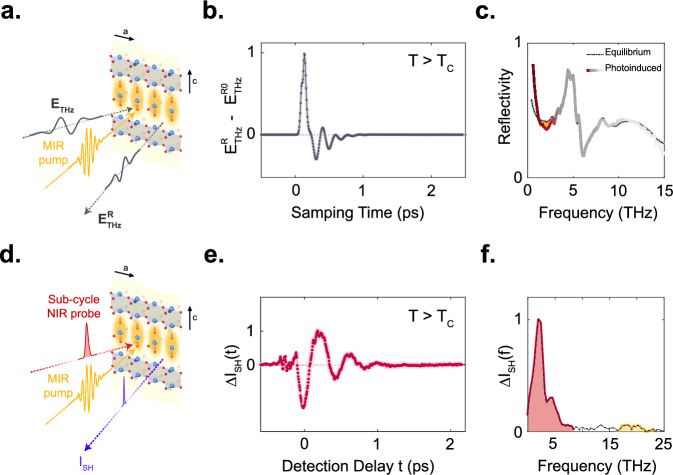
Light-induced Josephson plasmon dynamics. **a** Schematic of the mid-IR pump – THz probe experiment in YBa_2_Cu_3_O_6.48_. The sample is excited by a mid-IR pump pulse (yellow) polarized along the crystal c-axis, resonantly driving apical oxygen phonon modes as indicated inside the yellow shading. The subsequent changes in the low-frequency optical properties are sampled by a broadband, also c-polarized THz probe pulse (grey). **b** Photo-induced change in the reflected THz probe electric field at the peak of mid-IR pump-THz probe response, measured at a base temperature of 60 K (Tc = 48 K) **(c)** Sample reflectivity in equilibrium (dashed gray line) and following photo-excitation (solid dark red line at low frequencies <3 THz, solid grey line at frequencies above), measured at a base temperature of 60 K. The photo-induced changes are highlighted by yellow shading^6^. **d** Schematic of the mid-IR pump – time-resolved second harmonic probe experiment in the same sample. Here, the mid-IR pump pulse (yellow) is CEP stable and the subsequent dynamics are probed by collecting the second harmonic intensity (blue) generated from an 800 nm femtosecond probe pulse (red) as a function of detection delay t. Both incident pulses are polarized along the crystal c-axis. **e** Oscillatory contribution to the changes in the second harmonic intensity as a function of pump-probe time delay measured at T = 100 K, above T_c_. **f** Corresponding Fourier spectrum, highlighting the two resonantly excited apical oxygen phonons at 17 and 20 THz in yellow, and the low-frequency amplified Josephson plasmon in red^8^.

Resonant optical driving has been shown to induce transient optical properties reminiscent of superconductivity at temperatures above the transition temperature T_c_ in a range of materials, including certain molecular solids and cuprate compounds^1–11^. In underdoped YBa_2_Cu_3_O_6+x_, the effect is based on large-amplitude excitation of c-axis apical oxygen phonon modes using resonant mid-infrared pulses, inducing optical responses that are representatively shown in Fig. 1a–c. These optical properties include a 1/ω divergence in the imaginary part of the THz-frequency optical conductivity and a plasma edge in the reflectivity^2,6–8,12^.

Whether this short-lived non-equilibrium state has true microscopic features of a superconductor, that is a rigid condensate of Cooper pairs induced or stabilized by the optical driving, or if these effects are mainly connected to the nonlinear response of the material is still open to a definitive answer. Recent experiments have shown that this state expels magnetic fields, indicating an increase in mobility alone cannot explain these observations^13^.

The details of the mechanism underlying the observed state are not yet clarified. Not only is the analysis and interpretation of THz-frequency optical data a source of debate^14–24^, but also the theoretical efforts to conceptualize and extract a microscopic mechanism for the observed phenomena have not led to a conclusive set of hypotheses to be tested experimentally^25–30^. These challenges are also due to a lack of experimental data that directly reports on the microscopic non-equilibrium dynamics.

In YBa_2_Cu_3_O_6+x_, this phenomenon is connected to coherent dynamics of Josephson plasmon polaritons (JPPs), dispersive superconducting plasmons sustained by Cooper pair tunneling between the CuO_2_ planes^31–36^. Below *T*_*C*,_ where long-range superconducting coherence is well-formed throughout the crystal, JPPs are observed in equilibrium as weakly damped modes down to zero momentum. Above *T*_*C*_, in the so-called pseudo-gap phase, phase fluctuations cause JPPs to disappear at zero momentum, but they may still be present^37–44^ at finite momenta, supported by short-range phase fluctuating superconductivity. Whilst no zero-momentum JPP mode exists above *T*_*C*_, the phonon drive may parametrically couple to finite momentum JPPs both below and above *T*_*C*_.

These dynamics have recently been studied in single-pump time and angle-resolved (SHG) probe experiments^8^. The experimental schematic is shown in Fig. 1d. Concomitant with mid-infrared excitation, a 30-fs ultrafast NIR probe pulse of center wavelength 800 nm was incident on the sample to measure pump-induced SHG. Because equilibrium YBa_2_Cu_3_O_6+x_ is a centrosymmetric system, no (or very weak) static second harmonic is measured in absence of the pump. However, coherent motion of infrared-active modes, which dynamically break inversion symmetry as the mode coordinates oscillate about their equilibrium positions, result in a time-delay dependent SHG intensity $\varDelta {I}_{{SH}}$. The data of Fig. 1d were interpreted by proposing a three-mode mixing mechanism to justify the exponential amplification of low-frequency modes. The effect was discussed in terms of parametric mixing of a zone-center high frequency phonon, and two finite momentum JPPs. As will be discussed below, whilst the key elements of this interpretation are likely correct, the exact details of coupling are still not uniquely determined, leaving important aspects of the physics not fully clarified.

In this paper, a new form of multi-dimensional spectroscopy involving sequential excitation with two mid-infrared pulses and probing the SHG via a NIR pulse is used to further clarify the coherent dynamics following the phonon drive in YBa_2_Cu_3_O_6+x_. The new observations presented here point towards the parametric amplification of pairs of opposite-momentum JPPs via a four-mode (rather than three-mode) mixing process with the two resonantly-driven apical oxygen phonon modes. These amplified pairs possibly form a squeezed state, potentially akin to non-classical states proposed to reduce noise and redistribute fluctuations in photonic systems^45–48^.

We next turn to a detailed description of the basic theory for the experimental technique, revisit the evidence reported in ref. ^8^, and introduce the new multidimensional measurements.

## Results

### Time-resolved second harmonic generation probe

The coherent, time delay dependent oscillations in $\varDelta {I}_{{SH}}$ induced by an infrared-active mode ${Q}_{i}({\omega }_{i})$ of a solid at frequency ${\omega }_{i}$ can be cast in the stimulated hyper-Raman scattering formalism. This refers to a third-order nonlinear mixing, in which two photons of the probe field interact with the infrared-active mode to give rise to a hyper-Raman polarization ${P}_{i}(2{\omega }_{{pr}}\pm {\omega }_{i})=\,\frac{\partial {\chi }^{(2)}}{\partial {Q}_{i}}{Q}_{i}({\omega }_{i}){E}_{{pr}}^{2}({\omega }_{{pr}})$^49^. For a near-infrared probe at 800 nm wavelength $({\omega }_{{pr}}=375{THz}$), this interaction generates a sideband close to the second harmonic frequency of the probe at 400 nm (750 THz). Provided that the mode ${Q}_{i}({\omega }_{i})$ oscillates with a constant phase for all the laser shots in a pump-probe experiment and that the probe pulses are sufficiently short, the radiated hyper-Raman field ${E}_{i}(2{\omega }_{{pr}}\pm {\omega }_{i})$ can be measured as pump-probe-delay dependent oscillations of the SHG intensity $\varDelta {I}_{{SH}}$. In the absence of any other electromagnetic fields on the detector, one measures only time-delay dependent oscillations proportional to $\Delta {I}_{{SH},{Hom}} \sim {\left|{E}_{i}\right|}^{2}$, which occur at twice the mode frequency ${2\omega }_{i}$^50–53^. In the Nyquist sampling limit, this channel requires the probe pulses to be shorter than one quarter of the oscillation period of the mode ${Q}_{i}({\omega }_{i})$ and is known as *homodyne detection* $\Delta {I}_{{SH},{Hom}}$.

If interference with an auxiliary second-harmonic field takes place on the detector, a beating contribution $\Delta {I}_{{SH},{Het}} \sim {E}_{i}{E}_{{LO}}\cos \phi$ is detected between the auxiliary field (local oscillator ${E}_{{LO}}$) and the radiated hyper-Raman field ${E}_{i}$, leading to time delay dependent oscillations at the frequency of the mode ${\omega }_{i}$. This detection scheme relaxes the requirement on the probe pulse duration to half the period of the oscillating mode and is generally referred to as *heterodyne detection* $\Delta {I}_{{SH},{Het}}$. It was reported in ref. ^8^ that a spurious, time delay independent second harmonic field ${E}_{{LO}}$ at 400 nm wavelength (2${\omega }_{{pr}}$ = 750 THz), was co-propagating with the time-delay-dependent probe at the fundamental frequency ${\omega }_{{pr}}$. The presence of both homodyne and of a not-well controlled heterodyne response adds a degree of ambiguity when interpreting the one-dimensional data.

Representative data from ref. ^8^ are shown in Fig. 1e, f. The Fourier transform spectrum shows two peaks at about 17 and 20 THz (shaded in yellow) coinciding with the resonantly driven infrared-active apical oxygen phonons, which are present also in the linear response at small drive amplitudes. At the high drive fields, relevant for photo-induced superconductivity, the spectral response is dominated by a component near 2 THz, in the same frequency region where the photo-induced edge was observed in the THz reflectivity (Fig. 1c). The momentum and temperature dependence of this peak were also measured in ref. ^8^, and based on these results the peak was attributed to large-amplitude coherent oscillations of finite-momentum JPPs. Finally, the observed exponential scaling of the peak amplitude with the driven phonon suggests that the finite-momentum JPPs are being parametrically amplified.

### Model for nonlinear phonon-plasmon coupling

**Fig. 2:**
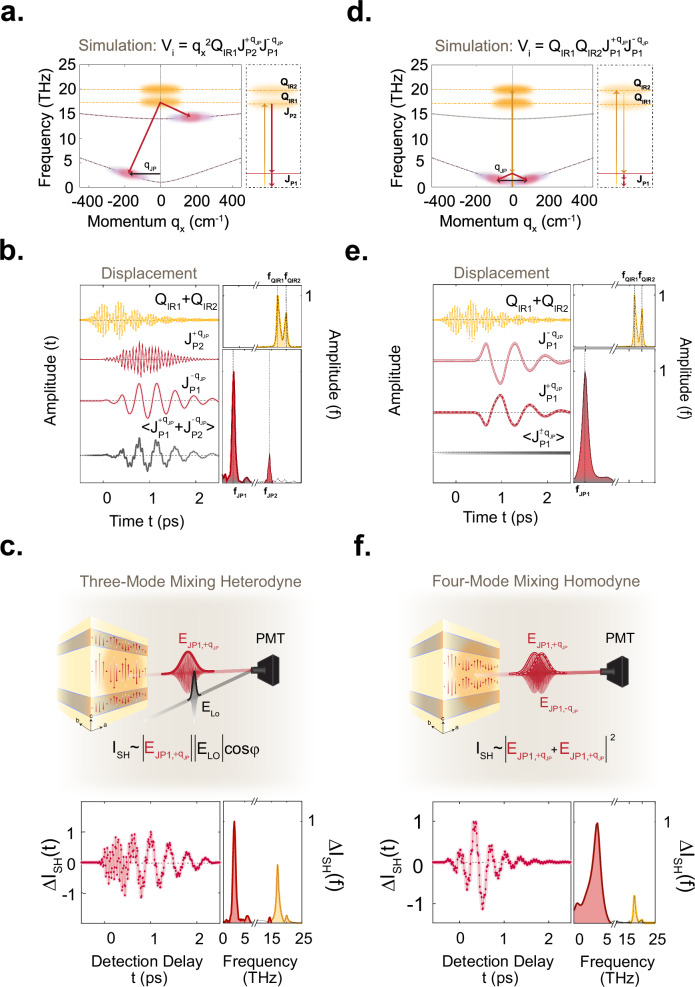
Comparison between the three and four-wave mixing models. **a** Left panel: dispersion curves of the two apical oxygen phonon modes (Q_IR1_ at 17 THz and Q_IR2_ at 20 THz) and of the inter-bilayer (J_P1_) and intra-bilayer (J_P2_) Josephson plasma polaritons along the in-plane momentum q_x_ are shown as yellow and red dashed lines, respectively. The mid-IR pump excites both apical oxygen phonon modes, which parametrically amplify a pair of inter-bilayer and intra-bilayer Josephson plasma polaritons at finite momentum q_JP_ (black arrow) such that ${\omega }_{{IR}1}={\omega }_{{JP}1}\left(-{q}_{{JP}}\right)+{\omega }_{{JP}2}({q}_{{JP}})$. The right panel depicts the energy level diagram corresponding to this model. **b** Time-dependent displacement of phonon modes, the two Josephson plasma polaritons and their average value following the photoexcitation, simulated using the three-mode mixing model (left panel) with their respective Fourier spectrum (right panel). The yellow shading indicates the two driven apical oxygen phonons while the two red shading is attributed to Josephson plasma polaritons. **c** Simulated oscillatory component of the changes in second harmonic intensity of the Josephson plasmons supercurrents in the heterodyne detection limit. Left panel: the time-delay dependent second harmonic intensity in the heterodyned detection limit following apical oxygen phonon excitation, simulated using the three-mode mixing model detailed in the text, and the corresponding Fourier spectrum using the same color shading as in (b)^8,54^. **d** Left panel: same dispersion relations as in (a) now for the four-mode mixing model explained in the text. The mid-IR pump again excites the two apical oxygen phonon modes. Now, they parametrically amplify a pair of inter-bilayer Josephson plasmon polaritons (J_P1_) at finite momentum $\pm {q}_{{JP}}$ (illustrated by the black arrow) such that ${\omega }_{{IR}2}-{\omega }_{{IR}1}=2{\omega }_{{JP}1}\left(\pm {q}_{{JP}}\right)$. The right panel shows the energy level diagram describing the four-mode mixing model. **e** Time-dependent displacement of phonon modes, the two Josephson plasma polaritons and their average value following the photoexcitation, simulated using the four-mode mixing model (left panel) with their respective Fourier spectrum (right panel). With the same color shading as in (b) and (c). **f** Simulated oscillatory component of the changes in second harmonic intensity of the Josephson plasmons supercurrents in the homodyne detection limit. From left to right: same as in (c) for four-mode mixing model but in the intermediate detection limit, using the same color shading as in (b), (c) and (e).

In ref. ^8^, the three-mode mixing process, sketched in Fig. 2a, was proposed to explain the observed large-amplitude coherent excitation of finite momentum JPPs (near 200 cm^-1^). This model describes a nonlinear parametric interaction between *only* one of the resonantly excited apical oxygen phonon modes (which have amplitudes denoted by ${Q}_{{IR}1}$ and ${Q}_{{IR}2}$ at frequencies ${\omega }_{{IR}1}/2\pi =$ 17 THz and ${\omega }_{{IR}2}/2\pi =$ 20 THz, respectively) with a pair of parametrically amplified finite-momentum (±${q}_{x}$) JPPs (with current coordinates ${J}_{P1}$ and ${J}_{P2}$ corresponding to the inter-bilayer and intra-bilayer tunneling modes at frequencies ${\omega }_{{JP}1}$ and ${\omega }_{{JP}2}$ respectively). For this effect to explain the one-dimensional data a Hamiltonian interaction term ${V}_{i}={\alpha }_{1}{q}_{x}^{2}{Q}_{{IR}1}{J}_{P2,{-q}_{x}}\,{J}_{P1,{q}_{x}}$ was proposed (see Supplementary Information). Figure 2c shows the result of numerical simulations based on this model carried out under the assumption of heterodyne detection, where coherent oscillations of the driven phonons and the coupled JPPs are detected at the modes’ eigenfrequencies. The calculations exhibit a close agreement to the experimental data.

As already recognized in ref. ^8^, and as shown in Fig. 2d–f, the data can also be described by an alternative parametric four-mode mixing coupling mechanism, which describes the interaction between *both* resonantly driven phonon modes ${Q}_{{IR}1}$ *and* ${Q}_{{IR}2}$, and fluctuating *pairs* of the lower frequency JPP in the form ${V}_{i}=\beta {({Q}_{{IR}1}+{Q}_{{IR}2})}^{2}\langle {J}_{P1,{q}_{x}}{J}_{P1,-{q}_{x}}\rangle$ (see Fig. 2d and ref. ^54^). Through the mixing term $\beta \left({Q}_{{IR}1}{Q}_{{IR}2}\right)\langle {J}_{P1,{q}_{x}}{J}_{P1,-{q}_{x}}\rangle$, it contains a resonance condition$\,{\omega }_{{squeezed}}=\,{\omega }_{{IR}2}-{\omega }_{{IR}1}$ for efficient amplification of fluctuating JPP pairs ${J}_{P1,{q}_{x}}$ and ${J}_{P1,{-q}_{x}}$ at frequency ${\omega }_{{JP}1}$, where ${\omega }_{{JP}1}\left(\pm {q}_{x}\right)=\frac{{\omega }_{{squeezed}}}{2}$ at identically-opposite momenta $\pm {q}_{x}$. This is especially interesting because it could lead to a squeezed state of the JPPs^55–57^.

Figure 2e shows the simulated dynamics of a pair of amplified plasmons, ${J}_{P1,{q}_{x}}$ and ${J}_{P1,{-q}_{x}}$, for a single laser shot, alongside the temporal phase-averaged response $\langle {J}_{P1,\pm {q}_{x}}\rangle$. Whilst ${J}_{P1,{q}_{x}}$ and ${J}_{P1,{-q}_{x}}$ are bound to respond coherently through the parametric process, the responses $\langle {J}_{P1,{q}_{x}}\rangle$ and $\langle {J}_{P1,-{q}_{x}}\rangle$, averaged over many pulses, are zero. In fact, the phase of the difference-frequency drive component ${Q}_{{IR}1}{Q}_{{IR}2}$ at frequency ${\omega }_{{IR}2}-{\omega }_{{IR}1}$ is phase-stable from shot to shot. Then, the phase of the parametrically amplified JPP pairs will be set to this absolute phase, however with a zero or a $\pi$ shift for different shots. As a result, after averaging over many excitation pulses in the experiment, the amplified plasmon responses $\langle {J}_{P1,{q}_{x}}\rangle$ and $\langle {J}_{P1,-{q}_{x}}\rangle$ remain zero. This is not the case for the product ${J}_{P1,{q}_{x}}{J}_{P1,-{q}_{x}}$, which has a fixed phase set by the ${Q}_{{IR}1}{Q}_{{IR}2}$ drive, hence the quantity $\langle {J}_{P1,{q}_{x}}{J}_{P1,-{q}_{x}}\rangle$ is non-zero.

Heterodyne SHG detection is blind to these dynamics. Although the generated currents ${J}_{P1,{q}_{x}}$ and ${J}_{P1,{-q}_{x}}$, when *taken individually*, are symmetry-odd, break the inversion symmetry and therefore emit two separate hyper-Raman fields ${E}_{{J}_{P1,+{q}_{x}}}(2{\omega }_{{pr}.}\pm {\omega }_{{J}_{P1,+{q}_{x}}})$ and ${E}_{{J}_{P1,-{q}_{x}}}(2{\omega }_{{pr}.}\pm {\omega }_{{J}_{P1,-{q}_{x}}})$, they cannot be detected in $\Delta {I}_{{SH},{Het}} \sim \left\langle {J}_{P1,{\pm q}_{x}}\right\rangle$ because the pulse averaged responses $\langle {J}_{P1,{q}_{x}}\rangle$ and $\langle {J}_{P1,-{q}_{x}}\rangle$ are zero. On the other hand, the product ${J}_{P1,{q}_{x}}{J}_{P1,-{q}_{x}}$, which oscillates at $2{\omega }_{{JP}1}$, is symmetry-even and not hyper-Raman active, hence does not modulate the SHG intensity (see Supplementary Information for details).

The situation changes if one considers homodyne detection. The measured intensity response of these fields from shot to shot is proportional to $\Delta {I}_{{SH},{Hom}} \sim {\left|{E}_{{J}_{P1,+{q}_{x}}}+{E}_{{J}_{P1,-{q}_{x}}}\right|}^{2}$ which contains the mixing terms $\sim \left\langle {E}_{{J}_{P1,+{q}_{x}}}{E}_{{J}_{P1,+{q}_{x}}}^{* }\right\rangle =\left\langle {E}_{{J}_{P1,-{q}_{x}}}{E}_{{J}_{P1,-{q}_{x}}}^{* }\right\rangle =\left\langle {E}_{{J}_{P1,\pm {q}_{x}}}{E}_{{J}_{P1,\mp {q}_{x}}}\right\rangle$. These terms generate homodyned SHG contributions, which yield a non-zero pulse-averaged intensity modulation at the frequency of the parametrically amplified plasmon oscillations ${2\omega }_{{JP}1}$. Figure 2f shows the results of simulating the single pump-SHG probe experiment using this four-mode mixing model, here carried out under the assumption of homodyne detection with a small heterodyne contribution. The results also show a very good agreement with the experimental data of Fig. 1e, f.

To summarize, the simulations of Fig. 2 demonstrate that the three-mode and four-mode mechanisms are indistinguishable in the single pump-probe experiment. For completeness, we also discuss in the Supplementary Information, how the three-mode mixing would impact on the homodyne detection scheme and how the four-mode mixing would affect the heterodyne detection, further underscoring the ambiguity in the earlier experiment.

### Two-dimensional nonlinear spectroscopy experiment

**Fig. 3:**
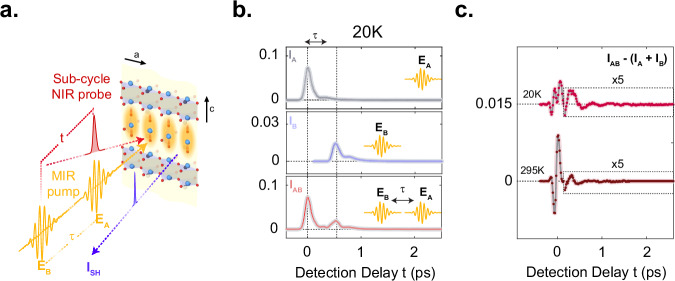
Two-dimensional mid-IR pump – time-resolved second harmonic generation probe. **a** Schematic of the same experiment as shown in Fig.1d, now using two CEP stable mid-IR pump pulses E_A_ and E_B_ separated by the excitation time delay τ with approximate fluences of 12 and 6 mJ/cm^2^ for ${E}_{A}$ and ${E}_{B}$, respectively. **b** From top to bottom: time-resolved SHG intensities ${I}_{A}$, ${I}_{B}$ and ${I}_{{AB}}$ measured in YBa_2_Cu_3_O_6.48_ at a base temperature of 20 K (below Tc), following excitation by only ${E}_{A}$, by only ${E}_{B}$ (after excitation delay τ) and by both pulses, respectively, at a base temperature of 20 K (below T_c_). **c** Nonlinear contribution to the time-resolved SHG intensity shown in panel (b), given as ${I}_{{AB}}-{I}_{A}-{I}_{B}$, and for base temperatures of 20 K and 295 K (below and above T_c_, respectively). The dashed rectangle frames the data at later time delays, which are enlarged by a factor of 5 for clarity.

In the present work this ambiguity was resolved by deploying a new form of two-dimensional spectroscopy^58–65^, based on a two-pump second-harmonic probe scheme with the aim of directly observing the inter-mode coupling processes responsible for the generation of coherent JPPs. The experimental setup is sketched in Fig. 3a. The two mid-infrared excitation pulses have electric fields denoted by *E*_*A*_ and *E*_*B*_. They resonantly drive the c-axis apical oxygen phonon modes at two instants in time, separated by a controllable time delay *τ*. The subsequent coherent dynamics of the JPP and phonon modes are then sampled by the near-infrared probe pulse at a time delay *t* (defined relative to the arrival time of the last excitation pulse, see Supplementary Information). The cooperative nonlinear contribution to the tr-SHG intensity ${I}_{{NL}}$ from both of the pump pulses is extracted by subtracting the isolated tr-SHG responses *I*_*A*_ and *I*_*B*_ (to only pulse *E*_*A*_ and *E*_*B*_, respectively), from the response *I*_*AB*_ (to both the excitation pulses): ${I}_{{NL}}={I}_{{AB}}-{I}_{A}-{I}_{B}$. Experimentally, *I*_*NL*_ is obtained by mechanically chopping the two excitation pulses at frequencies 1/2 and 1/3 of the laser repetition rate *f* and measuring the tr-SHG intensity component at their difference frequency *f*/6. This procedure is illustrated in Fig. 3b for an excitation pulse delay *τ* = 0.5 ps between *E*_*A*_ and *E*_*B*_. The individual tr-SHG signals *I*_*A*_ and *I*_*B*_ each contain a rectified response due to the third order nonlinear mixing of the electric filed of the pump with probe (electric-field induced SHG (EFISH)), which is then followed by coherent hyper-Raman responses of the driven phonons and amplified plasmons. After subtraction, *I*_*NL*_ reveals coherent dynamics due to nonlinear terms in the system Hamiltonian. This nonlinear response is also observed to be strongly dependent on temperature, as shown for two sample temperatures 20 K and 295 K, below and above the critical temperature *T*_*C*_, shown in Fig. 3c.

**Fig. 4:**
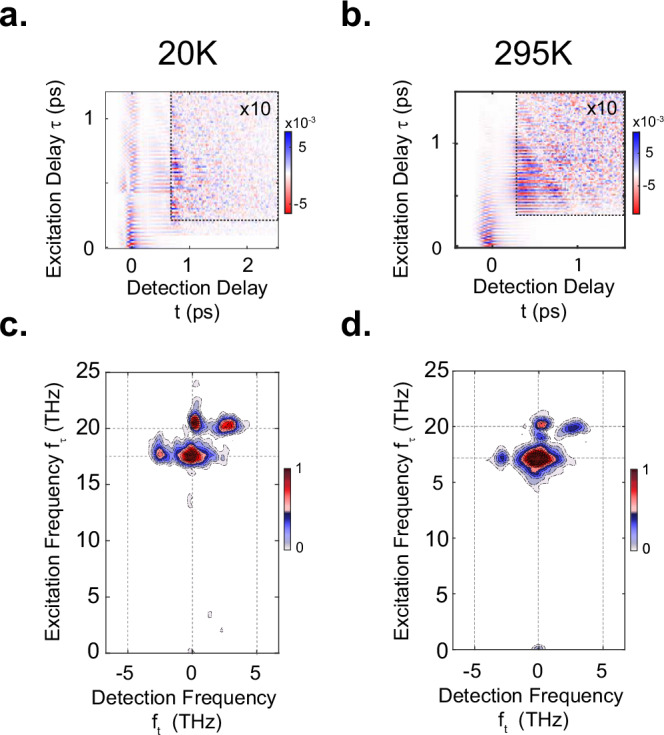
Two-dimensional nonlinear spectroscopy of the SHG response. **a** Nonlinear contributions to the time-resolved SHG intensity (as described in Fig.3) with the excitation time delay τ changing along the vertical axis, measured in YBa_2_Cu_3_O_6.48_ at a base temperature of 20 K (below T_c_). **b** Same as in (a) measured at 295 K (above T_c_). The data inside the black dashed box are multiplied by 10 for clarity. Data at both temperatures were measured with approximate fluences of 12 mJ. cm-2 and 6 mJ. cm-2 for EA and EB, respectively. **c** Normalized two-dimensional Fourier spectrum of the data inside the black dashed box in panel (a). **d** Same as (c) for the data shown in panel (b). Four strong peaks are found at frequency coordinates (0;17), (0_;_20), (-3_;_17), and (3_;_20), all in units of THz. The Fourier transformation for the two different temperatures are performed in different windows based on the time domain data and each 2D spectrum is normalized to its own maximum (see Supplementary Information).

Measurements of *I*_*NL*_ as a function of delay *τ* between the two mid-IR pump pulses yielded the two-dimensional time domain maps shown in Fig. 4a, b again for sample temperatures of 20 K and 295 K. For early *τ* and t, the rectified component of the homodyne contribution to the nonlinear SHG intensity dominates the response and masks the underlying coherent phonon-plasmon dynamics. Before Fourier transformation, with the goal of isolating the longer-lived oscillating signal component, the time-domain data were cropped along both time axes, indicated by the black dashed boxes in Fig. 4a, b, (see Supplementary Information). The corresponding two-dimensional Fourier spectra, which are symmetric around the origin, are shown in Fig. 4c, d for sample temperatures of 20 K and 295 K respectively. Each exhibit four dominant peaks. The two peaks at zero detection frequency, (*f*_*t*_,*f*_*τ*_) = (0;17) THz and (0;20) THz reflect homodyne-detected nonlinear tr-SHG in response to either of the two apical oxygen phonon modes *Q*_*IR1*_ and *Q*_*IR2*_ (as evidenced by their positions along the vertical f_*τ*_ axis). The two peaks at (-3;17) THz and (3;20) THz suggest that the dominant ~3 THz response observed in the pump-probe experiment of Fig. 1f (ref. ^8^) is driven cooperatively by the excitation of *both* apical oxygen phonon modes.

**Fig. 5:**
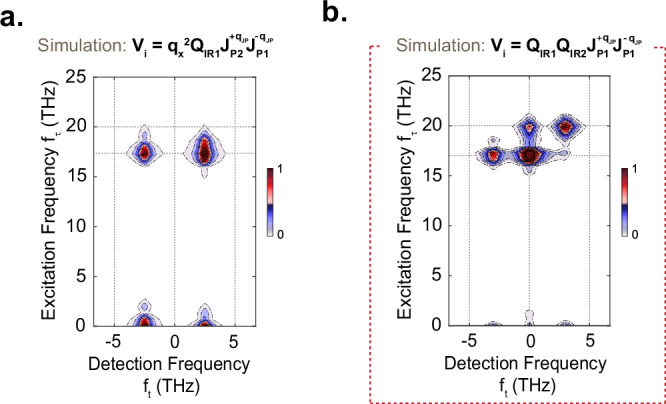
Comparison of the simulated nonlinear two-dimensional Fourier spectra according to three-wave and four-wave mixing models. **a** Simulated two-dimensional Fourier spectrum for the three-mode mixing model as outlined in Fig. 2b, showing four peaks at corresponding frequency coordinates (−2.5;17), (2.5;17), (−2.5;0) and (2.5;0), all in units of THz. **b** Simulated two-dimensional Fourier spectrum for the four-mode mixing model as outlined in Fig. 2d, with four strong peaks at corresponding frequency coordinates (0;17), (0;20), (−3;17) and (3;20), all in units of THz. The red dashed box emphasizes that the four-mode mixing model is compatible with experimental data shown in Fig. 4.

We next extend the simulations in Fig. 2 to calculate the corresponding multi-dimensional spectra, to identify the coupling mechanism leading to this peak pattern. For the three-mode mixing model with heterodyne detection, considered in ref. ^8^ and Fig. 2a–c, the resulting two-dimensional spectrum shown in Fig. 5a displays intense peaks at (−2.5;0) THz, (2.5;0) THz, (−2.5;17) THz and (2.5;17). These peaks arise from the nonlinear coupling between *only* the lower-frequency apical oxygen phonon *Q*_*IR1*_ and the two JPPs *J*_*P1*_ and *J*_*P2*_ (see Supplementary Information for details). Clearly, the simulated two-dimensional nonlinear spectrum does not match the measured spectrum, despite the agreement of the one-dimensional spectrum.

Figure 5b shows the two-dimensional spectrum resulting from the four-mode mixing model with homodyne detection, which also exhibited good agreement with the experiment in the one-dimensional spectrum. Here, we find four dominant peaks at (0;17) THz, (0;20) THz, (-3;17) THz and (3; 20) THz. All of these peaks include homodyne contributions of the two driven phonons (as their interference produces a difference-frequency response at 3 THz), and from their cooperative amplification of JPPs. As discussed earlier, the homodyne detection of the amplified JPPs retrieves the non-radiating covariance of the amplified Josephson plasmon pairs (see Supplementary Information for a detailed discussion). This peak pattern uniquely agrees with the experimental two-dimensional spectrum, thus resolving the ambiguity of the single-pulse pump-probe experiment.

**Fig. 6:**
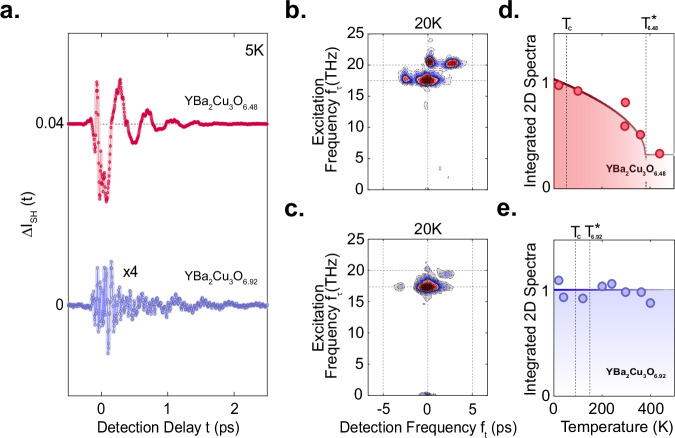
Temperature dependence of the SHG response in YBa_2_Cu_3_O_6.48_ and YBa_2_Cu_3_O_6.92_. **a** Oscillatory component of the Mid-IR pump induced changes in second harmonic intensity as a function of detection time delay t for YBa_2_Cu_3_O_6.48_ (red, T_c_ = 48 K, excitation fluence of 5 mJ.cm^-2^) and YBa_2_Cu_3_O_6.92_ (blue, T_c_ = 92 K, approximate excitation fluence of 43 mJ.cm^-2^ nearly eight times higher than the underdoped measurement), both at base temperature of 5 K (below T_c_). **(b)** Corresponding normalized nonlinear two-dimensional Fourier spectrum of YBa_2_Cu_3_O_6.48_ at base temperature of 20 K (below Tc), as in Fig. 4c. Excitation fluences for E_A_ and E_B_ were approximately 12 mJ.cm^-2^ and 6 mJ.cm^-2^, respectively. **c** Same as panel (b) for a different doping of YBa_2_Cu_3_O_6.92_. Excitation fluences for both E_A_ and E_B_ were approximately 8 mJ.cm^-2^. **d** Normalized frequency-integrated amplitude of nonlinear two-dimensional spectra of YBa_2_Cu_3_O_6.48_ as a function of base temperature (red circles, see Supplementary Information for details). The thick red line is a fit with a mean-field dependence (α + $\sqrt{1-{\rm{T}}/{{\rm{T}}}^{* }}$), indicating that this quantity has a dominant contribution that dereases as temperature approaches the pseudogap temperature T^*^ (380 K) and a contribution that takes a constant value α for all measured T. **e** Same as panel (d) for a different doping of YBa_2_Cu_3_O_6.92_. Here, the frequency-integrated nonlinear two-dimensional amplitude does not depend on temperature. Note that the temperature dependent data in each doping are measured under the same excitation fluence and the same two-dimensional time window for consistensy in the anaylysis.

We also carried out both single and two-pump-tr-SHG probe experiments in the higher-doped compound YBa_2_Cu_3_O_6.92_. At this doping, the apical oxygen phonon frequencies are the same as those of YBa_2_Cu_3_O_6.48_^37,66,67^, but the zero-momentum inter-bilayer Josephson plasma resonance ${{\rm{\omega }}}_{{\rm{JP}}1}$ is blue-shifted to 7 THz^68^. Figure 6a compares the coherent contributions to the single-pump probe tr-SHG intensity for YBa_2_Cu_3_O_6.48_ (T_c_ = 48 K) and YBa_2_Cu_3_O_6.92_ (T_c_ = 91 K) at a sample temperature of 5 K. Both curves include high-frequency oscillations of the driven phonons, while the lower-frequency oscillations are heavily suppressed in YBa_2_Cu_3_O_6.92_ compared to YBa_2_Cu_3_O_6.48_. Figure 6b, c show nonlinear two-dimensional spectra from YBa_2_Cu_3_O_6.48_ and YBa_2_Cu_3_O_6.92_, respectively (see Supplementary Information for the corresponding time-domain data). Although in both compounds the peaks appear at the same frequency positions, the relative amplitudes of the peaks are weaker in the YBa_2_Cu_3_O_6.92_ doping.

The two-dimensional temperature-dependent measurements in both compounds are shown in Figs. 6d, e. We find that the integrated 2D-peak amplitudes measured in YBa_2_Cu_3_O_6.48_ consist of two components. One is temperature-dependent, exhibits a mean-field dependence proportional to $\sqrt{1-T/{T}^{* }}$ with a characteristic temperature scale T* = 380 K, and is consistent with the temperature dependence reported in ref. ^8^, whilst a second component is weaker and temperature-independent. In contrast, the integrated 2D-peak amplitudes measured in YBa_2_Cu_3_O_6.92_ are temperature-independent up to 450 K, far above T^*^ = 160 K.

In the case of YBa_2_Cu_3_O_6.92_, there is no symmetry-odd mode below a frequency of 2 THz^66^ and the plasmon drive is far from resonance (${2{\rm{\omega }}}_{{\rm{JP}}1} > \,{{\rm{\omega }}}_{{\rm{IR}}2}-{{\rm{\omega }}}_{{\rm{IR}}1}$). Therefore, the data measured in YBa_2_Cu_3_O_6.92_ represents the 2D peak pattern which arises purely from homodyne mixing of the linearly excited phonon modes. Additionally, the 2D integrated amplitude being temperature independent implies that the excitation of the apical oxygen phonon modes is temperature independent as was also reported in ref. ^8^ based on the one-dimensional experiment.

We then attribute the temperature-dependent component of the 2D pattern in YBa_2_Cu_3_O_6.48_ to the four-mode mixing nonlinearity. Here, the plasmon amplification is a resonant process that dominates over the off-resonant temperature independent phonon homodyne mixing. The relevant temperature scale T* is consistent with the idea of finite frequency and momentum JPPs (${J}_{P1,\pm {q}_{x}}$) fluctuating throughout the pseudo-gap phase.

**Fig. 7:**
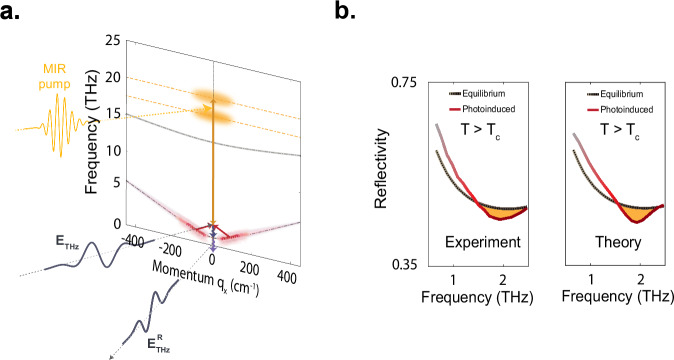
Four-mode mixing leading to photo-induced superconductivity in YBa_2_Cu_3_O_6.48_. **a** Illustration of the coupling between phonon-driven amplified Josephson plasmon polaritons, assuming the four-mode mixing model, and the THz probe field, resulting in the observed photo-induced reflectivity edge. The mid-IR excitation pulse (yellow) resonantly excites the two apical oxygen phonon modes ${Q}_{{IR}1}$ and ${Q}_{{IR}2}$ (yellow shading) which parametrically amplify pairs of inter-bilayer Josephson plasma polaritons ${J}_{P1}$ at frequencies ${\omega }_{{JP}1}({\pm q}_{{JP}})$ (red shading). These excitations renormalize the reflection coefficient, as measured by the THz probe field at q_x_ = 0 (grey pulses). **b** Comparison between experiment (left^2^,) and theory (right^54,69^,). Dashed black lines show the THz frequency reflectivity above T_c_ in equilibrium. Red solid lines are the THz frequency reflectivity following mid-IR excitation. In both plots, the yellow shaded area indicates the photo-induced changes at the photo-induced plasma edge (ω_JPLI_ < 2 THz).

The parametric plasmon amplification observed here provides a possible explanation for the measured superconducting-like features in the non-equilibrium THz reflectivity in YBa_2_Cu_3_O_6+x_. As illustrated in Fig. 7a, excitation of apical oxygen phonon modes at 17 THz and 20 THz leads to coherent amplification of pairs of finite momentum inter-bilayer JPPs which fulfill the resonance condition ${2\omega }_{{JP}1}(\pm {q}_{{JP}})=\,{\omega }_{{IR}2}-{\omega }_{{IR}1}$ at ${\omega }_{{JP}1}\approx$ 1.5 THz. These coherently amplified superconducting modes give rise to a characteristic plasma edge at ${\rm{q}}=0$, observed at a frequency blue-shifted relative to the equilibrium Josephson plasma resonance. A Fresnel-Floquet formalism was used to calculate the expected reflectivity of YBa_2_Cu_3_O_6.48_ under these driven conditions^54,69^ (see Supplementary Information). The results are shown in Fig. 7b (left panel). Starting from a featureless spectrum, a reflectivity edge emerges near 1.5 THz, in good agreement with experimental data (Fig. 7b (right panel))^2,6,54,69^.

## Discussion

In summary, multidimensional nonlinear spectroscopy was used to study the dynamics which follow strong-field excitation of the apical oxygen phonon modes and result in the emergence of superconducting-like transient THz frequency optical properties in YBa_2_Cu_3_O_6.48_. The 2D-peak pattern observed here, consisting of the cross-peak at ±3 THz, together with the photo-induced reflectivity edge at 1.5 THz, unambiguously point towards four-mode mixing between the two apical oxygen phonon modes and a pair of low-frequency modes. We note also that this four-mode mixing process possibly leads to the formation of a squeezed state of the lower frequency modes. While our results are consistent with a four-mode mixing by a pair of any optically active modes at 1.5 THz, the only candidates present in this frequency range below *T*_*C*_ are the Josephson plasma polaritons. The amplification of pairs of linearly dispersing acoustic phonons is ruled out, as they cannot be excited by symmetry, nor can they modulate the second harmonic. The T* temperature scale of the parametrically amplified state suggests that the pseudo-gap phase hosts finite frequency and finite momentum JPPs.

We note that the generation of squeezed Josephson plasmons may point towards a mechanism for phase stabilization or phase-noise reduction in an incoherent superconductor, to be further explored by future theoretical and experimental work. This draws a connection to the observation that a fluctuating pseudo-gap phase, hosting some form of phase-incoherent superconductivity, is a pre-requisite for the formation of non-equilibrium coherence. Indeed, the other material systems in which some form of light-induced enhancement of superconductivity has been observed, namely K_3_C_60_^4,9,11^ and κ-BEDT charge transfer salts^5,10^, also exhibit a strong vortex Nernst effect above Tc^70,71^. It remains to be understood if some form of mode-squeezing is relevant to the light-induced coherence in those compounds. More generally, the results reported here suggest a new framework for the engineering of parametrically amplified responses in materials, with potential connections to the physics of time crystal^72–75^ and to Floquet quantum matter^76,77^.

## Methods

### Sample preparation

The single crystals of YBa_2_Cu_3_O_6+δ_ were grown in Y-stabilized zirconium crucibles. The hole doping of the Cu-O planes was adjusted by controlling the oxygen content of the CuO chain layer through annealing in flowing O_2_ and subsequent rapid quenching. A DC magnetization measurement was carried out to determine the critical temperatures of the superconducting transitions for the two doping levels (T_c_ = 48 K for YBa_2_Cu_3_O_6.48_, and T_c_ = 91 K for YBa_2_Cu_3_O_6.92_). The single crystals of YBa_2_Cu_3_O_6.48_ and YBa_2_Cu_3_O_6.92_ ac-surfaces were polished and mounted into an optical cryostat with achievable temperature range of 5 to 450 K.

### Optical setup

In this experiment, a 1-kHz repetition rate Ti:sapphire femtosecond amplifier system (800 nm wavelength, 30 fs pulse duration) was used to pump two two-stage optical parametric amplifiers, seeded with the same white light continuum. The output signal pulses from the two OPAs, at 1235 nm and 1326 nm, were overlapped in a 350 μm thick GaSe crystal to generate CEP stable mid-IR pulses via the difference frequency generation (DFG). The mid-IR pump pulse duration (~150 fs duration) and frequency (~5 THz bandwidth, centered at 18 THz) were characterized by electro-optic sampling in a second GaSe crystal (~50 μm thickness). The mid-IR beam was focused to a spot size of ~ 70 μm on the sample. The pump polarization was fixed parallel to the YBa_2_Cu_3_O_6+x_ c-axis.

The pump-induced dynamics of the symmetry-odd modes in the YBa_2_Cu_3_O_6+x_ sample were sampled using time-resolved second harmonic generation of the 800 nm wavelength pulses, also polarized along the c-axis, which were focused to a spot diameter ~ 30 μm and overlapped with the mid-IR excitation pulses in a non-collinear geometry. The SH Intensity was collected in reflection geometry and detected by a photomultiplier.

The reflected probe pulses at 800 nm wavelength, used to detect the dynamics of symmetry-even modes, were separated from the SHG beam by a dichroic mirror. The photoinduced time-resolved polarization rotation of the 800 nm probe was measured by sending this beam to a half-wave plate and a Wollaston prism and detecting the difference signal of two intensity-balanced photodiodes.

In the nonlinear two-dimensional spectroscopy experiment, the excitation by two mid-IR pulses was accomplished by splitting the DFG output closely behind the GaSe crystal using a gold coated prism. One of the two pulses, E_A_, passed over a delay stage to control the excitation time delay τ and then recombined with E_B_ before being focused onto the sample. The spot diameters are of order ~100 μm in this setup. Both pulses E_A_ and E_B_ were individually characterized by electro-optic sampling. Both the time profile and the spectral content were not affected with respect to the single pulse used in the one-dimensional experiments.

The time-delay dependent second harmonic intensity was detected as detailed above for single-pulse excitation. To isolate the nonlinear contribution to the SH intensity, the two excitation pulses E_A_ and E_B_ were mechanically chopped at frequencies *f*_*Laser*_*/2* and *f*_*Laser*_*/3*, with *f*_*Laser*_ the laser repetition rate_,_ respectively. In this scheme, the nonlinear contribution appears at the difference frequency of the two choppers, i.e. at *f*_*Laser*_*/6*.

The time-delay dependent nonlinear polarization rotation of the 800 nm pulses was measured accordingly.

## Supplementary information

Supplementary Information

## Data Availability

The data that support the findings of this study are available from the corresponding author upon reasonable request.

## Code availability

The simulation codes used in this study are available from the corresponding author upon reasonable request.
