# Supplementary material for: Probing amplified Josephson plasmons in YBa2Cu3O6+x by multidimensional spectroscopy
Source: NPJ Quantum Mater. 2025 Jun 6;10(1):54. doi: 10.1038/s41535-025-00776-1 (PMC12141036; doi:10.1038/s41535-025-00776-1)
Supplement: Supplementary file 1 — Supplementary Information [file 41535_2025_776_MOESM1_ESM.pdf]

Probing amplified Josephson plasmons

in $YBa_2Cu_3O_{6+x}$ by multidimensional spectroscopy

N. Taherian[1], M. Först[1], A. Liu[1], M. Fechner[1], D. Pavicevic[1], A. von Hoegen[1], E. Rowe[1], Y. Liu[2], S. Nakata[2], B. Keimer[2], E. Demler[3], M. H. Michael[1], A. Cavalleri[1,4]

[1] *Max Planck Institute for the Structure and Dynamics of Matter, 22761 Hamburg, Germany*
[2] *Max Planck Institute for Solid State Research, 70569 Stuttgart, Germany*
[3] *Institute for Theoretical Physics, ETH Zurich, 8092 Zurich, Switzerland.*
[4] *Department of Physics, University of Oxford, Oxford OX1 3PU, United Kingdom*

Supplementary Information

Supplementary Note 1: Time-resolved second harmonic generation probe **2**

Supplementary Note 2: Modelling the driven phonon dynamics in $YBa_2Cu_3O_{6+x}$ **3**

Supplementary Note 3: Modelling the phonon-plasmon coupling in $YBa_2Cu_3O_{6.48}$ **7**

A. Three-mode mixing model **7**

B. Four-mode mixing model **11**

Supplementary Note 4: Squeezed plasmons dynamics **14**

Supplementary Note 5: Analytical model for nonlinear mode coupling **16**

Supplementary Note 6: Data Analysis **22**

A. Time-domain data for the single-pulse excitation **22**

B. Time-domain data for the two-dimensional spectroscopy **23**

C. Temperature dependence of the nonlinear two-dimensional spectrum **24**

Supplementary Note 7: THz reflectivity calculations within Fresnel-Floquet formalism **24**

Supplementary Note 8: Nonlinear phonon-phonon coupling in $YBa_2Cu_3O_{6.92}$ **25**

A.1. Single-pulse excitation experiment **26**

A.2. Two-dimensional nonlinear spectroscopy experiment **26**

B. Numerical simulation of the nonlinear phonon-phonon coupling in $YBa_2Cu_3O_{6.92}$ **27**

Supplementary Note 1: Time-resolved second harmonic generation probe

$YBa_2Cu_3O_{6+x}$ crystalizes in an inversion symmetric structure, therefore the second-order nonlinear optical susceptibility $\chi^{(2)}$ is zero. However, excitation with a mid-IR electric field $E_{pump}(t)$ launches coherent oscillations of symmetry-odd modes that transiently break the inversion symmetry to give rise to a time-dependent finite $\chi^{(2)}$ nonlinearity. This results in modulation of the second order nonlinear polarization as $P_i(t',t) = \frac{\partial\chi^{(2)}}{\partial Q_{IR}} Q_{IR}(t')E_{pr}^2(t'-t)$, where Q_{IR} represents the amplitude of any symmetry-breaking coordinate with frequency ω_{IR}, $\frac{\partial\chi^{(2)}}{\partial Q_{IR}}$ is the hyper-Raman coefficient of this mode (which we assume to be a constant within the probe spectral bandwidth), $E_{pr}(t'-t) = exp\left(-\frac{(t'-t)^2}{2\sigma_{pr}^2}\right) sin(\omega_{pr}(t'-t))$ with (σ_{pr}~30 fs, $\omega_{pr}/2\pi$~375 THz at 800 nm wavelength), t refers to the pump-probe time delay and t' refers to the lab time frame. This time dependent polarization emits an electric field $E_i(t',t)\ \alpha\ \frac{dP_i}{dt'}$ which contains frequency sidebands close to the 400-nm second harmonic of the probe light, i.e. at $(2\,\omega_{pr} \pm \omega_{IR})/2\pi$. The total nonlinear optical polarization is constructed together with the nonlinear polarization generated by the third-order nonlinear optical susceptibility $\chi^{(3)}$, which mixes the mid-IR pump with the probe electric field $P^{(3)}(t',t) \propto \chi^{(3)}\ E_{Pump}(t')\ E_{pr}^2(t'-t)$. Therefore, the total SH emitted field is $E_{SH} = \frac{d}{dt'}(P^{(3)} + P_i)$.

Experimentally, this response can be detected as the intensity[1-4]

$$I_{SH}(t) = A \int |E_{SH}(t',t) + E_{LO}(t')|^2\, dt' \tag{1.1}$$

$$= A\int\{|E_{SH}(t',t)|^2 + 2E_{Lo}(t')E_{SH}(t',t)cos\varphi + |E_{Lo}(t')|^2\}dt'$$

$$= I_{Hom}(t) + I_{Het}(t) + I_{bg} ,$$

where E_{LO} is the auxiliary reference electric field of a local oscillator and $I_{bg} = A\int |E_{Lo}(t')|^2 dt'$ its time-delay independent intensity at the detector.

In the absence of the local oscillator field $E_{LO} = 0$, the measured signal is sensitive to the intensity of only the emitted field, and the coherent oscillations of the mode Q_{IR} are detected at twice the fundamental frequency $(cos(2\omega_{IR}t))$. This detection scheme is known as *homodyne detection* limit, denoted as $I_{Hom}(t)$ in Eq. (1.1).

In the presence of an auxiliary local oscillator field $E_{LO} \neq 0$, its interference with the SH field generated in the sample results in a beating between the two. In this case, which is referred to as *heterodyne detection*, the detected intensity is proportional to $2E_{LO}E_{SH}cos\varphi$, where φ is the phase difference between the two fields. This term provides phase-sensitive detection of the Q_{IR} oscillations at the fundamental frequency ω_{IR} $(\sim sin(\omega_{IR}t))$ and is denoted as $I_{Het}(t)$ in Eq. (1.1).

Supplementary Note 2: Modelling the driven phonon dynamics in $YBa_2Cu_3O_{6+x}$

The $YBa_2Cu_3O_{6.92}$ compound hosts two IR-active, B_{1u}-symmetry apical oxygen phonon modes at frequencies $\omega_{IR1}/2\pi = 17$ THz and $\omega_{IR2}/2\pi = 20$ THz, which were resonantly driven to large amplitudes by c-axis polarized excitation pulses. The lattice potential, including the harmonic and an-harmonic terms of the driven modes and their coupling to the mid-IR electric field $E_{pump}(t)$ reads [5,6]

(2.1)

$$V_{phonons} = \frac{1}{2}(\omega_{IR1}^2 Q_{IR1}^2 + \omega_{IR2}^2 Q_{IR2}^2) + \frac{1}{4}(\kappa_{IR1} Q_{IR1}^4 + \kappa_{IR2} Q_{IR2}^4)$$

$$+(Z_{IR1}^* Q_{IR1} + Z_{IR2}^* Q_{IR2}) E_{pump}(t)\,.$$

Here, Q_{IR1} and Q_{IR2} represent the time dependent displacements of the two phonon modes, and κ_{IRi} and Z_i^* are their an-harmonic coefficients and mode effective charges, respectively.

The dynamics of the modes were calculated by numerically solving their equations of motion

(2.2)

$$\begin{cases} \ddot{Q}_{IR2} + 2\gamma_{IR2}\dot{Q}_{IR2} + \omega_{IR2}^2 Q_{IR2} = Z_{IR2}^* E - \kappa_{IR2} Q_{IR2}^3 \\ \ddot{Q}_{IR1} + 2\gamma_{IR1}\dot{Q}_{IR1} + \omega_{IR1}^2 Q_{IR1} = Z_{IR1}^* E - \kappa_{IR1} Q_{IR1}^3 \end{cases}$$

with γ_{IRi} being their phenomenological damping coefficients. The time-delay dependent second harmonic intensity $I_{SH}(t)$ connected to the oscillating phonon mode coordinates Q_{IR1} and Q_{IR2} was calculated along the equations described in Supplementary Note 1. The set of parameters used for these calculations is listed in the Supplementary Tables 1 and 2. The first table represents parameters used for the mid-IR excitation pulses and probe which were chosen to be comparable with the experimental values and are fixed for all the numerical simulations presented in this study. The parameters in the second table for the phonon modes are chosen within the range of the numbers extracted from DFT calculations. The remaining free parameters which are Hyper-Raman cross-sections for each individual mode were chosen such that the simulations fit the experimental data.

Supplementary Table 1: The set of parameters used for MIR pump and NIR probe in the numerical simulations discussed in this work.

	ω/2π (THz)	σ (fs)	E_0 (MV/cm)

Pump (E_A)	18.5	150	4
Pump (E_B)	18.5	150	12
Probe	375	30	-a

Supplementary Table 2: The set of parameters used in the numerical simulation of Eq. (2.2).

	$Z^*(e/\sqrt{u})$	ω/2π (THz)	γ (THz)	κ (meV/u^2A^4)
Q_{IR2}	0.3	20	1.7	0.8
Q_{IR1}	0.4	17	1.7	56

Note that at this doping level, the 17-THz mode has a larger oscillator strength compared to the 20 THz mode due to the larger percentage of filled Cu-O chains[7].

A.1. Single-pulse excitation

The electric field of a single mid-IR pump pulse is a sinusoid centred at frequency $\omega_{Pump}/2\pi = 18.5$ THz, multiplied by a Gaussian envelope of width $\sigma_{Pump} = 0.15$ ps

$$E(t) = E_0\, exp\left(-\frac{(t-t_0)^2}{2\sigma_{Pump}^2}\right) si\,n\left(\omega_{Pump}(t-t_0)\right). \tag{2.3}$$

Supplementary Figure 1 shows the solutions to the phonon equations of motion (Eq. (2.2)) for a peak electric field $E_0 = 12$ MV/cm and the resulting oscillatory contributions to the time-delay dependent SHG intensity for three different values of the local oscillator E_{LO}. A strong local oscillator allows these modes to be detected in the heterodyne SH intensity at their eigenfrequencies ω_{IR1} and ω_{IR2}. In the absence of a local oscillator E_{LO}, the two phonon modes are observed only in the homodyned SH intensity at twice their frequencies ($2\omega_{IR1}, 2\omega_{IR2}$), their sum frequency ($\omega_{IR1} + \omega_{IR2}$), all of which are outside our experimental time resolution, but also at their difference frequency ($\omega_{IR2} - \omega_{IR1}$). The

calculated SH intensities are compared to the experimental data in $YBa_2Cu_3O_{6.92}$, which were also shown in Fig. 6 (a) of the main text. Only the intermediate case of a small, but not negligible local oscillator provides agreement with the experiment.

A.2. Two-dimensional non-linear spectroscopy

In the two-pulse excitation experiment, the single mid-IR pulse was split into two electric fields E_A and E_B that are separated by the variable excitation time delay τ:

$$E_A(t) = E_{A0}\, exp\left(-\frac{(t-t_0)^2}{2\sigma_{Pump}^2}\right) sin(\omega_{Pump}(t-t_0)) \tag{2.4}$$

and

$$E_B(t+\tau) = E_{B0}\, exp\left(-\frac{((t+\tau)-t_0)^2}{2\sigma_{Pump}^2}\right) sin(\omega_{Pump}((t+\tau)-t_0))\,. \tag{2.5}$$

The total electric field $E_{AB}(t,\tau) = E_A(t) + E_B(t+\tau)$ is then used in the phonon equations of motion (Eq. (2.2)) to calculate the time dependent displacements of the two apical oxygen modes Q_{IR1} and Q_{IR2}.

Their nonlinear response to the mid-infrared excitation, measured as the cooperative nonlinear contribution from both of the pump pulses to the tr-SHG intensity I_{NL} as described in the main text, can equivalently be isolated in the simulations. First, the equations of motion were solved for excitation with only pulse E_A, only pulse E_B and with both excitation pulses E_{AB}. Then, the associated time-delay dependent changes in the second-harmonic intensity I_A, I_B and I_{AB} were calculated as described in Supplementary Note 1, and the nonlinear contribution obtained by subtracting the SHG intensity

responses to the individual excitation pulses from that to both excitation pulses as $I_{NL}(t,\tau) = I_{AB}(t,\tau) - I_A(t) - I_B(t,\tau)$.

For the case of $YBa_2Cu_3O_{6.92}$ and peak electric fields $E_{A0} = 4$ MV/cm and $E_{B0} = 12$ MV/cm, the simulated nonlinear two-dimensional SHG spectrum is shown in Supplementary Figure 2 for three different values of the reference field E_{LO}. For a strong local oscillator E_{LO} we find peaks only at detection frequencies that match the eigenfrequency of the driven phonon modes, resulting from their self-anharmonicities ($V_{nonl.} \sim Q_{IR,i}^4$). When E_{LO} is absent, we find a characteristic pattern that is dominated by the homodyne detection, yielding rectified components of the two driven modes at zero detection frequency and mixing components at their difference frequency. Comparison between these simulation results and the experimental data shown in Fig. 6 (a) and 6 (c) of the main text confirm the conclusion of Supplementary Note 2 that the experiment was carried out in the limit of a small reference field E_{LO}.

Supplementary Note 3: Modelling the phonon-plasmon coupling in $YBa_2Cu_3O_{6.48}$

According to References [8-11] and as discussed in the main text, the coupled phonon-plasmon dynamics detected in the single-pulse excitation experiments in $YBa_2Cu_3O_{6.48}$ can be interpreted by either a heterodyned SHG response of a three-mode mixing process or a homodyned SHG response of a four-mode mixing process. The nonlinear two-dimensional spectroscopy experiments presented here can differentiate between the two possible scenarios and favour the latter based on comparisons to simulation results of the underlying process. In this section, we discuss the details of these numerical simulations.

A. Three-mode mixing model

The three-mode mixing model describes the phonon-plasmon dynamics by mixing only one of the driven IR-active phonon modes with pairs of inter- and intra-bilayer Josephson plasmon polariton current coordinates, indicated by J_{P1} and J_{P2}, via a third order nonlinearity in the interaction potential. Simultaneous energy and momentum conservation define the resonance condition $\omega_{IR1} = \omega_{JP1}(-q_x) + \omega_{JP2}(q_x)$ for the frequencies and in-plane momenta $\pm q_x$ of the Josephson plasmon polaritons [8] (see Fig. 2(a) of the main text and Supplementary Figure 3(a)).

The excitation of the two IR-active apical oxygen phonons in $YBa_2Cu_3O_{6.48}$ is described by the same lattice potential presented for $YBa_2Cu_3O_{6.92}$ in Eq. (2.1). The driven nonlinear dynamics of the Josephson plasmon polaritons with harmonic potential $V_{plasmon} = \omega_{JP1}^2(q_x)J_{P1,q_x}J_{P1,-q_x} + \omega_{JP2}^2(q_x)J_{P2,q_x}J_{P2,-q_x}$ are determined by the interaction potential

(3.1)

$$V_{phonon-plasmon} = q_x^2\,(\alpha_1 Q_{IR1} + \alpha_2 Q_{IR2})\left(J_{P1,q_x}J_{P2,-q_x} + J_{P1,-q_x}J_{P2,q_x}\right),$$

where $J_{P1,\pm q_x}$ and $J_{P2,\pm q_x}$ represent the inter- and intra-bilayer plasmon current coordinates at frequencies $\omega_{JP1}(\pm q_x)$ and $\omega_{JP2}(\pm q_x)$, respectively. Here, q_x is the finite in-plane momentum and α_i are the phonon-plasmon coupling coefficients.

The time-delay dependent displacements of the phonon and current coordinates follow the coupled equations of motion

(3.2)

$$\begin{cases} \ddot{Q}_{IR2} + 2\gamma_{IR2}\dot{Q}_{IR2} + \omega_{IR2}^2 Q_{IR2} = Z_{IR2}^* E - \kappa_{IR2}Q_{IR2}^3 - \alpha_2 q_x^2 J_{P1,-q_x}J_{P2,q_x} - \alpha_2 q_x^2 J_{P1,-q_x}J_{P2,q_x} \\ \ddot{Q}_{IR1} + 2\gamma_{IR1}\dot{Q}_{IR1} + \omega_{IR1}^2 Q_{IR1} = Z_{IR1}^* E - \kappa_{IR1}Q_{IR1}^3 - \alpha_1 q_x^2 J_{P1,-q_x}J_{P2,-} - \alpha_1 q_x^2 J_{P1,-q_x}J_{P2,q_x} \\ \ddot{J}_{P2,q_x} + 2\gamma_{JP2}\dot{J}_{P2,q_x} + \omega_{JP2}^2(q_x)J_{P2,q_x} = -\alpha_1 q_x^2 Q_{IR1}J_{P1,-q_x} - \alpha_2 q_x^2 Q_{IR2}J_{P1,-q_x} \\ \ddot{J}_{P1,-q_x} + 2\gamma_{JP1}\dot{J}_{P1,-q_x} + \omega_{JP1}^2(-q_x)J_{P1,-q_x} = -\alpha_1 q_x^2 Q_{IR1}J_{P2,q_x} - \alpha_2 q_x^2 Q_{IR2}J_{P2,q_x} \end{cases}.$$

These equations were solved using a nonzero initial seed for the Josephson plasmon current coordinates. The optical excitation will parametrically amplify the plasmon coordinates with a phase set by the CEP stable pulses.

The set of parameters used to simulate the coupled phonon-plasmon dynamics following Eq. (3.7) are listed in the Supplementary Table 3. Here, the frequencies of the Josephson plasmon polaritons, $\omega_{JP1}(-q_x)/2\pi = 2.5$ THz and $\omega_{JP2}(q_x)/2\pi = 14.5$ THz, were set to fulfil the resonance condition for their amplification by the lower-frequency phonon Q_{IR1} at 17-THz frequency, in agreement with Reference [8]. The remaining free parameters which are the phonon-plasmon coupling coefficients and Hyper-Raman cross-sections for each individual mode were chosen such that the simulations fit the experimental data.

Supplementary Table 3: The set of parameters used in the numerical simulation of Eq. (3.2).

	$Z^*(\mathrm{e}/\sqrt{u})$	$\omega/2\pi$ (THz)	γ (THz)	κ (meV$/u^2A^4$)
Q_{IR2}	0.22	20	1.7	56
Q_{IR1}	0.25	17	1.7	56
J_{P2}	-	14.5	5	-
J_{P1}	-	2.5	2	-

A.1. Single-pulse excitation

The coupled equations were first solved for a single excitation pulse. Figure 2(b) of the main text and Supplementary Figure 3(b) show the time dependent displacements calculated from the simulated dynamics of the phonons $Q_{IR1}$and Q_{IR2} and the Josephson plasmon polaritons $J_{P1,-q_x}$ and J_{P2,q_X}. The corresponding oscillatory component of the time-resolved second harmonic intensity $I_{SH}(t)$ was then calculated according to the equations described in Supplementary Note 1. Note that the JPPs are symmetry-odd excitations and hence transiently modulate the second-harmonic, too (see also Reference 8).

Figure 2(c) of the main text and Supplementary Figure 3(c) and (d) summarize the results in the *heterodyne detection limit* (large amplitude of E_{LO}), together with their Fourier transformations. In this model, the phases of the current coordinates J_{P1} at 2.5 THZ and J_{P2} at 14.5 THz are fixed by the phase-stable optical excitation. Hence, the averaged response of these mode over many pump pulses is non-zero, and we find agreement with the experimental data presented in Figs. 1(e) and 1(f) of the main text.

For comparison, the SH intensity in the *homodyne detection limit* (small amplitude of E_{LO}) is shown in Supplementary Figure 3(e) and (f). Here, we find a 2.5 THz frequency component, arising from the aforementioned difference-frequency component of the driven phonons, along with a smaller component at 5 THz resulting from the homodyne contribution of the lower-frequency plasmon J_{P1} ($2\omega_{J_{P1}} = 5$ THz). The homodyne component of J_{P2} at 29 THz is outside the time resolution provided by the probe pulse duration, hence is not seen in Supplementary Figure 3(d).

In the main text, we only show the results calculated in the heterodyne detection limit (Figs. 2(a-c)), presented earlier in Ref. 8. Here, we find that both detection limits show a qualitative agreement with the experimental data, further emphasizing the ambiguity of the 1D experiment.

A.2. Two-dimensional nonlinear spectroscopy

To extend the numerical simulation for three-mode mixing model from the single-pulse to the two-pulse excitation, the coupled equations of motion shown in Eq. (3.7) were solved for a drive electric field $E_{AB}(t,\tau) = E_A(t) + E(t,\tau)$. The time-delay dependent nonlinear contribution to the second-harmonic intensity $I_{NL}(t,\tau) = I_{AB}(t,\tau) - I_A(t) - I_B(t,\tau)$ was then calculated following the equations outlined in Supplementary Note 1.

Supplementary Figure 4 summarizes the resulting nonlinear two-dimensional spectrum of the second-harmonic intensity for three different values of the reference field E_{LO}. In the main text, only Supplementary Figure 4 (c) was shown as Fig. 5 (a), because the discussion of the three-mode mixing model was restricted to the heterodyne detection limit.

Supplementary Figure 4 (c) shows that the nonlinear SHG response of the inter-bilayer Josephson plasmon at 2.5 THz is much larger compared to the one for the intra-bilayer Josephson plasmon and apical oxygen phonon modes and dominates the 2D spectrum. Importantly, based on the simulations shown here, in both detection limits the three-mode mixing model does not reproduce the peak pattern measured in the experiment as shown in Fig. 4 of the main text.

B. Four-mode mixing model

In the four-mode mixing model of the coupled phonon-plasmon dynamics, statistical fluctuations of the Josephson plasmon coordinates are parametrically amplified cooperatively by *both* apical oxygen phonon modes, Q_{IR1} and Q_{IR2}, through a forth-order nonlinearity with resonance condition $2\omega_{JP1}(\pm q_x) = \omega_{IR2} - \omega_{IR1}$. In the following, we discuss the details of the simulations performed for the single-pulse and two-pulse excitation experiments.

We model the coupled dynamics by the same phonon potential V_{phonon} introduced in Eq. (2.1) and an additional phonon-plasmon interaction that now reads

(3.3)

$$V_{phonon-plasmon} = \beta(Q_{IR1} + Q_{IR2})^2 J_{P1,q_x} J_{P1,-q_x} \,.$$

Here, the resonantly driven apical oxygen phonons parametrically excite pairs of finite-momentum Josephson plasmon polaritons J_{P1,q_x} and $J_{P1,-q_x}$ via a force proportional to

$(Q_{IR1} + Q_{IR2})^2$. The subscript q_x refers to this in-plane momentum and β is the coupling coefficient.

The corresponding coupled equations of motion for the Josephson plasmon polaritons are

$$\begin{cases} \ddot{Q}_{IR2} + 2\gamma_{IR2}\dot{Q}_{IR2} + \omega_{IR2}^2 Q_{IR2} = Z_2^* E - \kappa_{IR2} Q_{IR2}^3 - \beta (Q_{IR1} + Q_{IR2})^2 J_{P1,q_x} J_{P1,-q_x} \\ \ddot{Q}_{IR1} + 2\gamma_{IR1}\dot{Q}_{IR1} + \omega_{IR1}^2 Q_{IR1} = Z_1^* E - \kappa_{IR1} Q_{IR1}^3 - \beta (Q_{IR1} + Q_{IR2})^2 J_{P1,q_x} J_{P1,-q_x} \\ \ddot{J}_{p1,q_X} + 2\gamma_{JP1}\dot{J}_{P1,q_x} + \omega_{JP1}^2(q_x) J_{P1,q_x} = -\beta (Q_{IR1} + Q_{IR2})^2 J_{P1,q_x} \\ \ddot{J}_{p1,-q_X} + 2\gamma_{JP1}\dot{J}_{P1,-q_x} + \omega_{JP1}^2(q_x) J_{P1,-q_x} = -\beta (Q_{IR1} + Q_{IR2})^2 J_{P1,-q_x} \end{cases} \tag{3.4}$$

We solved these equations of motion with a seed of random initial phase for the plasmon coordinates. Still, the parametric force for the plasmon coordinates, which is the difference frequency component of the $Q_{IR1}Q_{IR2}$ term, dictates the phase of the amplified plasmons to be either π or zero.

The set of parameters used to simulate the phonon-plasmon dynamics following the Eq. (3.9) are listed in the Supplementary Table 4. The plasmon frequencies were set by the above specified resonance condition. The remaining free parameters which are the phonon-plasmon coupling coefficients and Hyper-Raman cross-sections for each individual mode were chosen such that the simulations fit the experimental data.

Supplementary Table 4: The set of parameters used in the numerical simulation of Eq. (3.4).

	$Z^*(\mathrm{e}/\sqrt{u})$	$\omega/2\pi$ (THz)	γ (THz)	κ (meV/u^2A^4)
Q_{IR2}	0.33	20	1.3	56
Q_{IR1}	0.34	17	1.5	56
J_{P1,q_x}	-	1.5	2.2	-
$J_{P1,-q_x}$	-	1.5	2.2	-

B.1. Single-pulse excitation

The coupled equations were first solved for a single-pulse excitation. The total time dependent displacement of the phonons Q_{IR1} and Q_{IR2} and the Josephson plasmon polaritons $J_{P1,q_x}$and $J_{P1,-q_x}$ are shown in Fig. 2(e) of the main text and in Supplementary Figure 5(b). The amplified plasmons follow either the light-dotted red or the dark-dashed curves, where we have shown only one initial phase. Averaging over the random initial phases results in $\langle J_{P1}^{\pm q_x} \rangle = 0$. The corresponding time-delay dependent oscillatory contributions to the second harmonic intensity $I_{SH}(t)$ were again calculated along the formalism described in Supplementary Note 1 for both, the heterodyne and homodyne detection limits.

The SH intensity calculated in the *heterodyne detection limit* is shown in Supplementary Figure 5 (c) and (d). As explained above, the coordinates of the Josephson plasmon polaritons average to zero ($\langle J_{P1}^{+q_x} \rangle = \langle J_{P1}^{-q_x} \rangle = 0$), and hence cannot be detected, in contrast to the coherent dynamics of the driven phonon modes. Comparing this calculation to the measured 1D data (Figs. 1(e), 1(f) of the main text), this response does not provide a good agreement.

On the other hand, Fig. 2(f) of the main text together with Supplementary Figure 5(f) illustrate the results in the homodyne detection limit with only small value for E_{LO}. The time domain data and the Fourier transformations show a good agreement with the experimental data presented in Figs. 1(e) and 1(f) of the main text. In this limit, the homodyne mixing of the plasmon terms proportional to $\langle J_{P1,q_x} J_{P1,-q_x} \rangle$, described in detail in the main text, will not average to zero and overlaps with the homodyne beating contribution of the driven phonon modes at 3 THz.

In the main text, the discussion was restricted to the homodyne detection limit, and Supplementary Figure 5 (a), (b), (e) and (f) were shown as Fig. 2(d-f).

B.2. Two-dimensional non-linear spectroscopy

The simulations of the coupled phonon-plasmon dynamics via the four-mode mixing model were extended to the case of the two-pulse excitation. We used the same procedure described in Supplementary Note 3.A.2., now with the equations of motion for the four-mode mixing model shown in Eq. (3.9). Supplementary Figure 6 shows the resulting nonlinear two-dimensional spectrum of the time-resolved SHG intensity for different values of E_{LO}.

The nonlinear two-dimensional SHG spectrum of this four-mode mixing model successfully reproduces the peak pattern detected in the two-pulse excitation experiment in $YBa_2Cu_3O_{6.48}$, which is not the case for the spectrum simulated within the three-mode mixing model. Moreover, from the detailed discussion in Supplementary Note 3.B. and the two-dimensional simulations, we conclude that the parametric amplification of the Josephson plasmon via the four-mode mixing results in an enhancement of the previously discussed phononic peak pattern considered in the limit of a small local oscillator E_{LO} (see Supplementary Note 2 and Supplementary Figure 2).

Supplementary Note 4: Squeezed plasmons dynamics

As discussed in the main text, the beating of the fluctuating plasmons $\langle J_{P1,q_x} J_{P1,-q_x} \rangle$ forms a symmetry-even, non-radiating squeezed state which oscillates at $2\omega_{JP1}(\pm q_x)$. In this section we discuss the dynamics of this squeezed mode from a theoretical point of view. To this end, we rewrite the phonon-plasmon interaction potential for Eq. (3.8) as

(4.1)

$$V_{phonon-plasmon} = \sum_{q_x} P_{P1,q_x} P_{P1,-q_x} + \left(\omega_{JP1}^2 + c^2 q_x^2\right) J_{P1,q_x} J_{P1,-q_x} + \beta (Q_{IR1} + Q_{IR2})^2 J_{P1,q_x} J_{P1,-q_x} ,$$

where P_{P1,q_x} is the generalized plasmon momentum.

The corresponding coupled equations of motion derived from the Ehrenfest equations ($\partial_t O = i[H, O]$) for the Josephson plasma fluctuations are

(4.2)

$$\partial_t \left(J_{P1,q_x} J_{P1,-q_x}\right) = P_{P1,-q_x} J_{P1,q_x} + P_{P1,q_x} J_{P1,-q_x}$$

(4. 3)

$$\partial_t \left(P_{P1,-q_x} J_{P1,q_x} + P_{P1,q_x} J_{P1,-q_x}\right) = -2\left(\omega_{JP1}^2 + c^2 q_x^2\right) J_{P1,q_x} J_{P1,-q_x} - 2\beta (Q_{IR1} + Q_{IR2})^2 J_{P1,q_x} J_{P1,-q_x} + 2 P_{P1,q_x} P_{P1,-q_x}$$

(4.4)

$$\partial_t \left(P_{P1,q_x} P_{P1,-q_x}\right) = -\left(\omega_{JP1}^2 + c^2 q_x^2\right)\left(P_{P1,-q_x} J_{P1,q_x} + P_{P1,q_x} J_{P1,-q_x}\right) - \beta (Q_{IR1} + Q_{IR2})^2 \left(P_{P1,-q_x} J_{P1,q_x} + P_{P1,q_x} J_{P1,-q_x}\right)$$

The time dependent plasma response is obtained by first linearizing the driven current coordinate and momentum fluctuations around their equilibrium values

(4.5)

$$\langle J_{P1,q_x} J_{P1,-q_x} \rangle_{drive} = \langle J_{P1,q_x} J_{P1,-q_x} \rangle - \langle J_{P1,q_x} J_{P1,-q_x} \rangle_{equil}$$

(4.6)

$$\langle P_{P1,q_x} P_{P1,-q_x} \rangle_{drive} = \langle P_{P1,q_x} P_{P1,-q_x} \rangle - \langle P_{P1,q_x} P_{P1,-q_x} \rangle_{equil} ,$$

which are related to each other by

$$\partial_t\langle J_{P1,q_x}J_{P1,-q_x}\rangle_{equil} = \partial_t\langle P_{P1,q_x}P_{P1,-q_x}\rangle_{equil} = \partial_t\,\langle P_{P1,q_x}J_{P1,-q_x} + J_{P1,q_x}P_{P1,-q_x}\rangle_{equil}$$
$$= Q_{IR1} = Q_{IR2} = 0\,. \tag{4.7}$$

These conditions lead to the relations $\langle P_{P1,q_x}J_{P1,-q_x} + J_{P1,q_x}P_{P1,-q_x}\rangle_{equil} = 0$ and $\langle P_{P1,q_x}P_{P1,-q_x}\rangle_{equil} = \frac{1}{\omega_{JP1}^2+c^2q_x^2}\langle J_{P1,q_x}J_{P1,-q_x}\rangle_{equil}$, finally resulting in the equation of motion for the phonon-driven Josephson plasma polariton fluctuations

$$\left(\partial_t^2 + 4\left(\omega_{JP1}^2 + c^2q_x^2\right)\right)\langle J_{P1,q_x}J_{P1,-q_x}\rangle = -2\beta(Q_{IR1} + Q_{IR2})^2\langle J_{P1,q_x}J_{P1,-q_x}\rangle_{equil}\,, \tag{4.8}$$

which gives a resonant response when $2\omega_{JP1}(\pm q_x) = \omega_{IR2} - \omega_{IR1}$ (= 3 THz considered here) for an in-plane momentum given by $c^2q_{JP}^2 \approx \frac{(\omega_{IR2}^2-\omega_{IR1}^2)^2}{4} - \omega_{JP1}^2$. This equation shows that the equilibrium fluctuations are crucial for the four-mode mixing model. Additionally, it shows that the squeezing dynamics are resonantly excited at $2\omega_{JP1}(\pm q_{JP})$ via the $Q_{IR1}Q_{IR2}$ force and with a phase that is fixed by the phase difference of the two phonons $\varphi_{Q_{IR1}Q_{IR2}} = \varphi_{Q_{IR2}} - \varphi_{Q_{IR1}}$.

From Eq. (2.1), and assuming initial thermal fluctuations $\langle J_{P1,q_x}J_{P1,-q_x}\rangle_{equil}$, we can trace back the non-radiating oscillations of $\langle J_{P1,q_x}J_{P1,-q_x}\rangle$ at $2\omega_{JP1}$ from the SHG measurement in the homodyne detection limit. This intuition is confirmed in the numerical simulations which include dissipation and random phase fluctuations[11].

Supplementary Note 5: Analytical model for nonlinear mode coupling

To develop a deeper understanding of the one- and two-dimensional Fourier transformations in both the experiments and the numerical simulations, we developed a perturbative expansion approach to solve the equations of motion analytically. First, we

introduce this methodology for a single resonantly excited mode with forth-order nonlinearity and then extend it to the case of the two driven apical oxygen phonon modes in $YBa_2Cu_3O_{6.92}$.

We consider the lattice potential already described in Eq. (2.1). For a single IR-active mode Q_i with resonance frequency ω_i, mode effective charge Z_i^* and forth order self-anharmonicity κ_i, driven by an excitation pulse electric field $E(t)$, it reads

$$V_i = \frac{1}{2}\omega_i^2 Q_i^2 + \frac{1}{4}\kappa_i Q_i^4 + Z_i^* Q_i E \tag{5.1}$$

The equation of motion for this mode is

$$\ddot{Q}_i + 2\gamma_i \dot{Q}_i + 2\omega_i^2 Q_i = -Z_i^* E - \kappa_i Q_i^3, \tag{5.2}$$

where the damping coefficient γ_i was introduced.

In the perturbative expansion approach, the drive electric field $E(t)$ is replaced by $\lambda E(t)$, where λ changes continuously from zero to unity and determines the strength of the interaction (λ will be set to one at the end).

$$\ddot{Q}_i + 2\gamma_i \dot{Q}_i + 2\omega_i^2 Q_i = -Z_i^* \lambda E - \kappa_i Q_i^3 \tag{5.3}$$

We can seek solutions to this equation in the form of a power series of λ

$$Q_i = \lambda Q_i^{(1)} + \lambda^2 Q_i^{(2)} + \lambda^3 Q_i^{(3)} + \cdots \tag{5.4}$$

and rewrite Eq. (5.3) as

(5.5)

$$\left(\lambda\ddot{Q}_i^{(1)}+\lambda^2\ddot{Q}_i^{(2)}+\lambda^3\ddot{Q}_i^{(3)}\right)+2\gamma_i\,(\lambda\dot{Q}_i^{(1)}+\lambda^2\,\dot{Q}_i^{(2)}+\lambda^3\,\dot{Q}_i^{(3)})+\omega_0^2(\lambda Q_i^{(1)}+\lambda^2 Q_i^{(2)}+\lambda^3 Q_i^{(3)})=\,-\lambda Z_i^* E-\kappa_i {Q_i^{(1)}}^3$$

Next, all the terms in the equation above can be reorganized proportional to the powers of λ and separated into equations (5.6–5.8)

(5.6)

$$\ddot{Q}_i^{(1)}+2\gamma_i\dot{Q}_i^{(1)}+\omega_i^2 Q_i^{(1)}=\,-\lambda Z_i^* E$$

(5.7)

$$\ddot{Q}_i^{(2)}+2\gamma_i\dot{Q}_i^{(2)}+\omega_i^2 Q_i^{(2)}=\,0$$

(5.8)

$$\ddot{Q}_i^{(3)}+2\gamma_i\dot{Q}_i^{(3)}+\omega_i^2 Q_i^{(3)}=\,-\kappa_i {Q_i^{(1)}}^3$$

Using this approach, the linear and nonlinear responses are represented by $Q_i^{(1)}$ and $Q_i^{(n)}$ for n > 1, respectively. Hereafter, we exemplarily discuss the driven phonon dynamics in $YBa_2Cu_3O_{6.92}$ following single and two-pulse excitation.

A.1. Single-pulse excitation

For a delta function electric field $E(t) = E_0\delta(t)$, the first order impulsive response of the driven damped harmonic oscillator $Q_i^{(1)}$ can be written as

(5.9)

$$Q_i^{(1)}(t)=\begin{cases}0 & , \quad t<0\\ -\dfrac{Z_i^* E_0}{\Omega_i}e^{-\gamma_i(t)}\,sin(\Omega_i t) & , \quad t\geq 0\end{cases}$$

with $\Omega_i = \sqrt{\omega_i^2 - \gamma_i^2}$.

The third-order correction is obtained by substituting $Q_i^{(1)}(t)$ into Eq. (5.8) and finding the analytical solution

(5.10)

$$-\kappa_i {Q_i^{(1)}}^3 = -\kappa_i \left(-\frac{Z_i^* E_0}{\Omega_i} e^{-\gamma_i(t)} \sin(\Omega_i t)\right)^3$$

$$= \frac{Z_i^* \kappa_i E_0}{\Omega_i} e^{-3\gamma_i(t)} \left(\frac{3}{4} \sin(\Omega_i t) - \frac{1}{4} \sin(3\Omega_i t)\right).$$

These two equations show that the linear and non-linear responses contain a term resonating at the fundamental and another term resonating at the third harmonic frequency.

According to Eq. (5.9), Q_{IR1} and Q_{IR2} develop as

(5.11)

$$Q_{IR1}(t, \tau) = E'_{IR1} e^{-\gamma_{IR1}(t)} \sin\left(\Omega_{IR1}(t)\right)$$

and

(5.11)

$$Q_{IR2}(t, \tau) = E'_{IR2} e^{-\gamma_{IR2}(t)} \sin\left(\Omega_{IR2}(t)\right) ,$$

with $\Omega_{IRi} = \sqrt{\omega_{IRi}^2 - \gamma_{IRi}^2}$ and $E'_{IRi} = -\frac{Z_{IRi}^* E_0}{\Omega_{IRi}}$.

The time-delay dependent SHG intensity is again calculated following the equations presented in Supplementary Note 1. In the limit of homodyne detection with $I_{Hom} \sim |\sum_i E_i|^2$, the signal contains frequency components at $(\Omega_{IR1} + \Omega_{IR2})$ and $(\Omega_{IR2} -$

Ω_{IR1}). In the heterodyne detection, the contributions resulting from the interference term I_{Het} ~ $|E_i||E_{LO}|cos\varphi$ appear at the phonon modes eigenfrequencies Ω_{IR1} and Ω_{IR2}.

These contributions to the analytical solution are confirmed by the numerical simulation results shown in Supplementary Figure 2. The peak at $(\Omega_{IR1} + \Omega_{IR2})$ is beyond the resolution of the experiment and hence cannot be observed.

A.2. Two-dimensional non-linear spectroscopy

Next, we extend these results for the same Hamiltonian but with two pulses exciting the system. Once again, for simplicity, the excitation pulses are in the form of delta functions $E_{AB}(t,\tau) = E_{0A}\delta(t) + E_{0B}\delta(t+\tau)$.

Following excitation with both pulses, the first order response can be rewritten as

(3.12)

$$Q_i^{(1)}(t,\tau) = Q_{iA}^{(1)}(t) + Q_{iB}^{(1)}(t,\tau)$$

$$= -\frac{Z_i^* E_{0A}}{\Omega_i} e^{-\gamma_i(t)} \, sin(\Omega_i t) \; - \frac{Z_i^* E_{0B}}{\Omega_i} e^{-\gamma_i(t+\tau)} \, sin\big(\Omega_i(t+\tau)\big) \, ,$$

where $Q_{iA}^{(1)}(t)$ is the first order response to only the excitation pulse E_A and $Q_{iB}^{(1)}(t,\tau)$ is the first order response to only the excitation pulse E_B. The total response in the second harmonic intensity is then obtained by incorporating the time-resolved probing scheme explained in Supplementary Note 1 to the response of the coordinates Q_{IR1} and Q_{IR2}.

For the two-pulse excitation, we again restrict the analytical approach to only the first order response

(5.13)

$$Q_{IRi,AB}(t,\tau\,) = E'_{IRi} e^{-\gamma_{IRi}(t)} \, sin\big(\Omega_{IRi}(t)\big) + E'_{IRi} e^{-\gamma_{IRi}(t+\tau)} \, sin\big(\Omega_{IRi}(t+\tau)\big) \, .$$

For simplicity we assumed that $E_{0A} = E_{0B}$.

The time-delay dependent SHG intensity associated with these dynamics is again calculated following the equations presented in Supplementary Note 1, considering the two-pulse excitation approach of Supplementary Note 2.A.2.

As the perturbative solution is restricted to only the first order response, which is linear to the driving electric field, the nonlinear contribution to the SHG intensity in the heterodyne detection limit is zero. In contrast, there is a nonzero response in the nonlinear contribution in the homodyne detection limit, because it is sensitive to mixing terms from the two phonon fields. Equation (5.17) and (5.18) show the mathematical terms obtained using the analytical approach to the first order response.

$$I_{NL}^{het} = I_{AB}^{het} - (I_A^{het} + I_B^{het}) = 0 \tag{5.17}$$

$$I_{NL}^{hom} = I_{AB}^{hom} - (I_A^{hom} + I_B^{hom}) = \tag{5.18}$$

$$\left\{ \begin{array}{c}
2E''_{IR1,A}\, E''_{IR1,B}\Omega_{IR1}^2 e^{-\gamma_{IR1}(2t+\tau)}\, cos(\Omega_{IR1}t)\, cos(\Omega_{IR1}(t+\tau)) \\
-2\, E''_{IR1,A}\, E''_{IR1,B}\Omega_{IR1}\gamma_{IR1} e^{-\gamma_{IR1}(2t+\tau)}\, sin(\Omega_{IR1}t)\, cos(\Omega_{IR1}(t+\tau)) \\
-2E''_{IR1,A}\, E''_{IR1,B}\Omega_{IR1}\gamma_{IR1} e^{-\gamma_{IR1}(2t+\tau)}\, sin(\Omega_{IR1}(t+\tau))\, cos(\Omega_{IR1}t) \\
+2E''_{IR1,A}\, E''_{IR1,B}\gamma_{IR1}^2 e^{-\gamma_{IR1}(2t+\tau)}\, sin(\Omega_{IR1}t)\, sin(\Omega_{IR1}(t+\tau)) \\
+2E''_{IR1,A}\, E''_{IR2,B}\Omega_{IR1}\Omega_{IR2} e^{-\gamma_{IR1}t} e^{-\gamma_{IR2}(t+\tau)}\, cos(\Omega_{IR1}t)\, cos(\Omega_{IR2}(t+\tau)) \\
+2E''_{IR2,A}\, E''_{IR1,B}\Omega_{IR1}\Omega_{IR2} e^{-\gamma_{IR1}(t+\tau)} e^{-\gamma_{IR2}t}\, cos(\Omega_{IR2}t)\, cos(\Omega_{IR1}(t+\tau)) \\
-2E''_{IR1,A}\, E''_{IR2,B}\Omega_{IR1}\gamma_{IR2} e^{-\gamma_{IR1}t} e^{-\gamma_{IR2}(t+\tau)}\, sin(\Omega_{IR2}(t+\tau))\, cos(\Omega_{IR1}t) \\
-2E''_{IR2,A}\, E''_{IR1,B}\Omega_{IR1}\gamma_{IR2} e^{-\gamma_{IR1}(t+\tau)} e^{-\gamma_{IR2}t}\, sin(\Omega_{IR2}t)\, cos(\Omega_{IR1}(t+\tau)) \\
-2E''_{IR1,A}\, E''_{IR2,B}\Omega_{IR2}\gamma_{IR1} e^{-\gamma_{IR1}t} e^{-\gamma_{IR2}(t+\tau)}\, sin(\Omega_{IR1}t)\, cos(\Omega_{IR2}(t+\tau)) \\
-2E''_{IR1,B}\, E''_{IR2,A}\Omega_{IR2}\gamma_{IR1} e^{-\gamma_{IR1}(t+\tau)} e^{-\gamma_{IR2}t}\, sin(\Omega_{IR1}(t+\tau))\, cos(\Omega_{IR2}t) \\
+2E''_{IR1,A}\, E''_{IR2,B}\gamma_{IR1}\gamma_{IR2} e^{-\gamma_{IR1}t} e^{-\gamma_{IR2}(t+\tau)}\, sin(\Omega_{IR1}t)\, sin(\Omega_{IR2}(t+\tau)) \\
+2E''_{IR2,A}\, E''_{IR1,B}\gamma_{IR1}\gamma_{IR2} e^{-\gamma_{IR1}(t+\tau)} e^{-\gamma_{IR2}t}\, sin(\Omega_{IR2}t)\, sin(\Omega_{IR1}(t+\tau)) \\
+2E''_{IR2,A}\, E''_{IR2,B}\Omega_{IR2}^2 e^{-\gamma_{IR2}(2t+\tau)}\, sin(\Omega_{IR2}t)\, cos(\Omega_{IR2}(t+\tau)) \\
-2E''_{IR2,A}\, E''_{IR2,B}\Omega_{IR2}\gamma_{IR2} e^{-\gamma_{IR2}(2t+\tau)}\, sin(\Omega_{IR2}t)\, cos(\Omega_{IR2}(t+\tau)) \\
-2E''_{IR2,A}\, E''_{IR2,B}\Omega_{IR2}\gamma_{IR2} e^{-\gamma_{IR2}(2t+\tau)}\, sin(\Omega_{IR2}(t+\tau))\, cos(\Omega_{IR2}t) \\
+2E''_{IR2,A}\, E''_{IR2,B}\gamma_{IR2}^2 e^{-\gamma_{IR2}(2t+\tau)}\, sin(\Omega_{IR2}t)\, sin(\Omega_{IR2}(t+\tau))
\end{array} \right.$$

The sixteen terms in this solution give rise to twelve observable peaks in the nonlinear 2D spectrum, with frequency coordinates (f_t;f_τ); (($\Omega_{\mathrm{IR2}} - \Omega_{\mathrm{IR1}}$); Ω_{IR2}), (($\Omega_{\mathrm{IR1}} - \Omega_{\mathrm{IR2}}$);$-\Omega_{\mathrm{IR2}}$), (($\Omega_{\mathrm{IR1}} - \Omega_{\mathrm{IR2}}$); Ω_{IR1}), (($\Omega_{\mathrm{IR2}} - \Omega_{\mathrm{IR1}}$);$-\Omega_{\mathrm{IR1}}$), (0; $\pm\,\Omega_{\mathrm{IR2}}$) and (0; $\pm\,\Omega_{\mathrm{IR1}}$). In addition, there are eight more peaks ($\pm$(Ω_{IR1}+ Ω_{IR2}); $\pm\,\Omega_{\mathrm{IR1}}$), (($\pm$($\Omega_{\mathrm{IR1}}$+ Ω_{IR2}); $\pm\,\Omega_{\mathrm{IR2}}$), (2$\Omega_{\mathrm{IR1}}$; $\pm\,\Omega_{\mathrm{IR1}}$) and (2$\Omega_{\mathrm{IR2}}$; $\pm\,\Omega_{\mathrm{IR2}}$) which, due to the probe time resolution, are resolved in neither the numerical simulations nor the experiment. This analytically calculated peak pattern, arising from only phonon mode dynamics, is consistent with both the numerically simulated and experimentally measured 2D spectra of $YBa_2Cu_3O_{6.92}$, as shown in Supplementary Figure 7.

In this treatment, the first-order response of the phonon mode coordinates results in a nonzero contribution to the nonlinear SHG intensity only in the homodyne detection whilst the heterodyne detection is zero. Nonlinear responses in the heterodyne detection limit, as for example shown in the numerically simulated two-dimensional spectra in Supplementary Figure 2, occur only if higher-order responses, such as the forth order nonlinearities of the driven phonons, are taken into account.

Supplementary Note 6: Data Analysis

A. Time-domain data for the single-pulse excitation

The coherent oscillations in the tr-SHG intensity $\Delta I_{SH}(t)$ were isolated by subtracting the slowly varying background, resulting from the third order nonlinear mixing between the mid-IR pump and the near infrared probe at time zero in the material (proportional to $\chi^{(3)}E_{pump}E_{pr}^2$). To this end, we fitted the following fitting function to the raw data which was subsequently subtracted.

$$\Delta I_{SH} = \sum_i A_i \left(1 + erf\left(\frac{(t-t_0)}{\sigma_i}\right)\right) \cdot exp\left(\frac{-(t-t_0)}{\tau_i}\right) \quad (6.1)$$

Supplementary Figure 8 shows examples of this procedure for the data plotted in Figs. 1 (e) and 6 (a) of the main text.

B. Time-domain data for the two-dimensional spectroscopy

For qualitative discussion on the peak pattern arising from coherent dynamics, the time domain data shown in Figs. 4 and 6 (a) and (d) were cropped. This was done because nonlinear signal at early time delays along the detection delay t-axis consists of large rectified component of the homodyne contributions of the individual modes (as explained in Supplementary Notes 1 and 5) as well as mixing terms proportional to the electric fields of E_A and E_B excitation pulses to which dominates the longer-lived oscillatory response resulting from the nonlinear mixing of modes. In particular, each pump pulse electric field results in a nonlinear polarization $P_i(2\omega_{pr} \pm \omega_{pump}) = \chi^{(3)} E_i(\omega_{pump}) E_{pr}^2(\omega_{pr})$ which radiate two fields $E_{A,emit}$ and $E_{B,emit}$ (the so-called electric field induced second harmonic (EFISH)) as a result of E_A and E_B, respectively. In the second harmonic homodyne detection measurement, these fields are measured as $I_{SH,A} = \left|E_{A,emit}\right|^2$for only pulse E_A, $I_{SH,AB} = \left|E_{B,emit}\right|^2$for only pulse E_B and $I_{SH,AB} = \left|E_{A,emit} + E_{B,emit}\right|^2$ for when both excitation pulses E_{AB} are exciting the sample - neglecting all the other symmetry breaking contributions. From this the nonlinear second harmonic intensity can be derived as $I_{SH,NL} = I_{SH,AB} - I_{SH,A} - I_{SH,B} \approx E_{A,emit} E_{B,emit}$. Similarly, the homodyne SHG component of the nonlinear mixing between the resonantly excited apical oxygen phonon modes dominates along the excitation axis τ. Hence, to distil the nonlinear plasmon-phonon coupling, we cropped the time-domain data simultaneously along both time axes t and τ,

using a smooth error function $\left(1 + erf\left(\frac{(S-S_1)}{S_2}\right)\right)$, prior to the Fourier transformation. Here, S represents the time axis (t or τ), S_1 and S_2 represent the cropping time and rise time of the error function (being different for each time axis), respectively. For all of the analysis done here, S_2 has been set to be 0.1 ps.

Supplementary Figure 9 (a) and (b) display the uncropped 2D-time map for measurements below and above T_c at 20 K and 295K, respectively. Exemplary 2D-time domain data cropping procedure including the raw and cropped data taken in $YBa_2Cu_3O_{6.48}$ at base temperature 20 K (below T_c) along with their corresponding nonlinear 2D spectra are shown in Supplementary Figure 9 (c-e).

Supplementary Figure 10 illustrates the same procedure for $YBa_2Cu_3O_{6.92}$ at base temperature 20 K (below T_c). The corresponding 2D spectrum is also shown in the main text Fig. 6 (c).

C. Temperature dependence of the nonlinear two-dimensional spectrum

As discussed in the main text, the peaks in the nonlinear 2D spectra of $YBa_2Cu_3O_{6.92}$ arise from phonon dynamics only, while the phonon-plasmon coupling adds additional intensity to the spectra in $YBa_2Cu_3O_{6.48}$. To distinguish the two contributions, the temperature dependencies of the two doping levels, shown in Figs. 6 (d) and 6(e) of the main text, were obtained for each temperature by integrating the amplitudes in the raw 2D Fourier spectrum along both frequency axes.

Supplementary Note 7: THz reflectivity calculations within Fresnel-Floquet formalism

The THz reflectivity at equilibrium and after mid-IR excitation, shown in Fig. 7 (b) of the main text, were calculated using the Fresnel-Floquet approach outlined in References [9,10], where the oscillations at the difference frequency $\omega_F = \omega_{IR2} - \omega_{IR1}$ are discussed to produce a Floquet medium. In the signal and idler basis, the eigenstates and eigenvalues of the Floquet medium are found using the basis

$$\begin{pmatrix} \epsilon_\infty\left(\omega^2\omega_{JP}^2\right) + i\frac{\sigma_{DC}}{\epsilon_0}\omega - c^2q^2 & A \\ A & \epsilon_\infty\left((\omega-\omega_F)^2 - \omega_{JP}^2\right) + i\frac{\sigma_{DC}}{\epsilon_0}(\omega-\omega_F) - c^2q^2 \end{pmatrix} \cdot \begin{pmatrix} E(\omega) \\ E(\omega-\omega_F) \end{pmatrix} = 0 \quad (7.1)$$

Here, ω is the frequency of the probe beam, $\omega - \omega_F$ is the idler frequency, q is the in-plane momentum, E(ω) is the electric field, ϵ_∞ is the background material permittivity, $\frac{\sigma_{DC}}{\epsilon_0}$ is a dissipation term resulting from the quasiparticle contribution to the conductivity, c is the speed of light and A captures the amplitude of the $Q_{IR1}Q_{IR2}$ oscillations. The reflectivity was computed using the Fresnel-Floquet approach [9,10] with parameters $\frac{\omega_F}{2\pi}$ = 3.8 THz, A = 37.6 (THz)^2, $\frac{\sigma_{DC}}{\epsilon_0}$ = 36 THz, $(\frac{\omega_{JP}}{2\pi})^2$= 0.37 (THz)^2 and ϵ_∞ = 25.

Supplementary Note 8: Nonlinear phonon-phonon coupling in $YBa_2Cu_3O_{6.92}$

In $YBa_2Cu_3O_{6+x}$, the resonantly driven apical oxygen phonon modes $Q_{IR1}$and Q_{IR2} also couple nonlinearly to symmetry-even A_g Raman modes $Q_{R,i}(\omega_{R,i})$ via a third-order nonlinear interaction[8,12,13]. Coherent oscillations of these Raman mode produce a nonlinear optical polarization $P_i(\omega_{pr} \pm \omega_{R,i}) = \frac{\partial\chi^{(1)}}{\partial Q_{R,i}} Q_{R,i}(\omega_{R,i})E_{pr}(\omega_{pr})$, frequency-shifted from the incoming probe field at 800 nm wavelength ($\omega_{pr}/2\pi = 375\ THz$), which

can be measured as a time-delay dependent polarization rotation of the probe. In the following, we discuss measurements and simulations of this nonlinear phonon coupling, which yields responses distinct from those of the symmetry-breaking modes presented above.

A.1. Single-pulse excitation experiment

Supplementary Figure 11(a) displays a sketch of the mid-IR pump, infrared polarization-rotation probe experiment and Supplementary Figure 11(b) a typical time-delay dependent response measured in $YBa_2Cu_3O_{6.92}$ at room temperature. Slowly varying contributions to the response were subtracted beforehand similarly to the approach described for the time-resolved second-harmonic intensity in Supplementary Note 6.A.

The coherent phonon oscillations were fitted using the following function

$$F_{fit} = \sum_i A_{R,i}\left(1 + \mathrm{erf}\left(\frac{(t-t_0)}{\tau_{R,i}}\right)\right)\sin(\omega_{R,i}t)\exp\left(\frac{t-t_0}{\gamma_{R,i}}\right), \tag{8.1}$$

where A_i, τ_i, $\omega_{R,i}$ and $\gamma_{R,i}$ indicate the amplitudes, rise times, frequencies and decay times of the Raman modes, and t_0 is time zero. The Fourier transforms of the data and of the numerical fit, shown in Supplementary Figure 11 (c), reveal a superposition of coherent oscillations of three fully symmetric A_g phonon modes at frequencies 3.6, 4,4 and 5.1 THz.

A.2. Two-dimensional nonlinear spectroscopy experiment

The same experiment was then conducted with a pair of mutually delayed excitation pulses in order to extract the nonlinear interaction between the resonantly driven symmetry-odd and nonlinearly coupled symmetry-even modes. Similar to the second-harmonic probe experiments, this nonlinear contribution $\Delta E_{PR,NL}$ was obtained by subtracting the coherent response to excitation pulses only E_A and only E_B ($\Delta E_{PR,A}$ and

$\Delta E_{PR,B}$) from the response $\Delta E_{PR,AB}$ to the both excitation pulses E_{AB}: $\Delta E_{PR,NL} = \Delta E_{PR,AB} - \Delta E_{PR,A} - \Delta E_{PR,B}$.

Supplementary Figure 12 (a) and (b) show a sketch of the experimental setup, together with the nonlinear signal $\Delta E_{PR,NL}$ for an excitation delay $\tau = 0.045$ ps, again measured at room temperature in $YBa_2Cu_3O_{6.92}$. The corresponding two-dimensional time-delay dependent nonlinear polarization rotation map and the Fourier spectrum of the indicated black dashed box are shown in Supplementary Figure 12 (c) and (d). The nonlinear two-dimensional frequency map reveals four strong peaks at frequency coordinates (f_t, f_τ); (0 THz, 17 THz), (0 THz, 20 THz) and a weaker peak at (17 THz, -3.6 THz). This 2D spectrum illustrates the nonlinear coupling between the resonantly driven infrared-active apical oxygen phonons and the Raman-active modes.

B. Numerical simulation of the nonlinear phonon-phonon coupling in $YBa_2Cu_3O_{6.92}$

In this section, we use numerical simulations to model the time resolved polarization rotation experiments in $YBa_2Cu_3O_{6.92}$ and to develop further insight into the third-order nonlinear phonon-phonon coupling. The resonant excitation of the two infrared-active apical oxygen phonons is described by the same lattice potential discussed in Eq. (2.1). The nonlinear coupling of these modes to Raman-active phonon modes $Q_{R,i}(\omega_{R,i})$ with harmonic potentials $V_i = \frac{1}{2}\omega_{R,i}^2 Q_{R,i}^2$ is determined by the known interaction potential

(8.2)

$$V_{phononics} = \sum_i (g_i Q_{IR1}^2 + h_i Q_{IR2}^2) Q_{R,i} ,$$

where g_i and h_i account for the nonlinear coupling coefficients. The corresponding coupled equations of motion for all the phonon modes are

(8.3)

$$\begin{cases} \ddot{Q}_{IR2} + 2\gamma_{IR2}\dot{Q}_{IR2} + \omega_{IR2}^2 Q_{IR2} = Z_2^* E - \kappa_{IR2} Q_{IR2}^3 \\ \ddot{Q}_{IR1} + 2\gamma_{IR1}\dot{Q}_{IR1} + \omega_{IR1}^2 Q_{IR1} = Z_1^* E - \kappa_{IR1} Q_{IR1}^3 \\ \ddot{Q}_{R,1} + 2\gamma_{R,1}\dot{Q}_{R,1} + \omega_{R,1}^2 Q_{R,1} = -g_1 Q_{IR1}^2 - h_1 Q_{IR2}^2 \\ \ddot{Q}_{R,2} + 2\gamma_{R,2}\dot{Q}_{R,2} + \omega_{R,2}^2 Q_{R,2} = -g_2 Q_{IR1}^2 - h_2 Q_{IR2}^2 \\ \ddot{Q}_{R,3} + 2\gamma_{R,3}\dot{Q}_{R,3} + \omega_{R,3}^2 Q_{R,3} = -g_3 Q_{IR1}^2 - h_3 Q_{IR2}^2 \end{cases}$$

with $\gamma_{R,i}$ the phenomenological damping coefficients. The Q_{IRi}^2 quadratic force on the Raman modes $Q_{R,j}$ predicts the latter to be rectified along their coordinates and to oscillate at their eigenfrequencies around the transient displacements [12,14].

The time-delay dependent probe polarization rotation, induced by the three Raman phonon mode coordinates $Q_{R,i}$, was calculated as

(8.4)

$$\Delta E_i(t) = A \int \frac{\partial \chi^{(1)}}{\partial Q_{R,i}} Q_{R,i}(t') E_{pr}(t' - t)\, dt'$$

The set of parameters used for calculating the phonon dynamics from Eq. (8.3) is listed in the table below, with the Raman mode frequencies taken from the measurements shown above. The remaining free parameters which are the phonon-phonon coupling coefficients and Raman cross-sections for each individual mode were chosen such that the simulations fit the experimental data.

Supplementary Table 5: The set of parameters used in the numerical simulation of Eq. (8.3).

Label	$Z^*(\mathrm{e}/\sqrt{u})$	$\omega/2\pi$ (THz)	γ (THz)	κ (meV/u^2A^4)
Q_{IR2}	0.3	20	1	0.8
Q_{IR1}	0.4	17	1	56
$Q_{R,1}$	-	3.6	1	-
$Q_{R,2}$	-	4.4	1	-
$Q_{R,3}$	-	5.1	1	-

B.1. Single-pulse excitation numerical simulation

The coupled equations were first solved for a single-pulse excitation. Supplementary Figure 13 (a) and (b) show the simulated $\Delta E_{PR} = \sum_i \Delta E_i(t)$ for the three A_g Raman-active modes and the corresponding Fourier spectrum, respectively. The simulations result shows a good agreement with the experimental data shown in Supplementary Figure 12 (b) and (c).

B.2. Two-dimensional nonlinear spectroscopy numerical simulation

To reproduce the two-pulse excitation, the coupled equations of motion in Eq. (8.3) were solved for a drive electric field $E_{AB}(t,\tau) = E_A(t) + E_B(t,\tau)$. The time-delay dependent nonlinear contribution to the time-resolved polarization rotation was then calculated following the procedure outlined above $\Delta E_{PR,NL} = \Delta E_{PR,AB} - \Delta E_{PR,A} - \Delta E_{PR,B}$.

Supplementary Figure 13 (c) summarizes the nonlinear two-dimensional spectrum simulated using the nonlinear phonon-phonon interaction potential of Eq. (8.2). It successfully reproduces the experimental 2D spectrum and confirms the third-order nonlinear phonon-phonon coupling.

SUPPLEMENTAL FIGURES

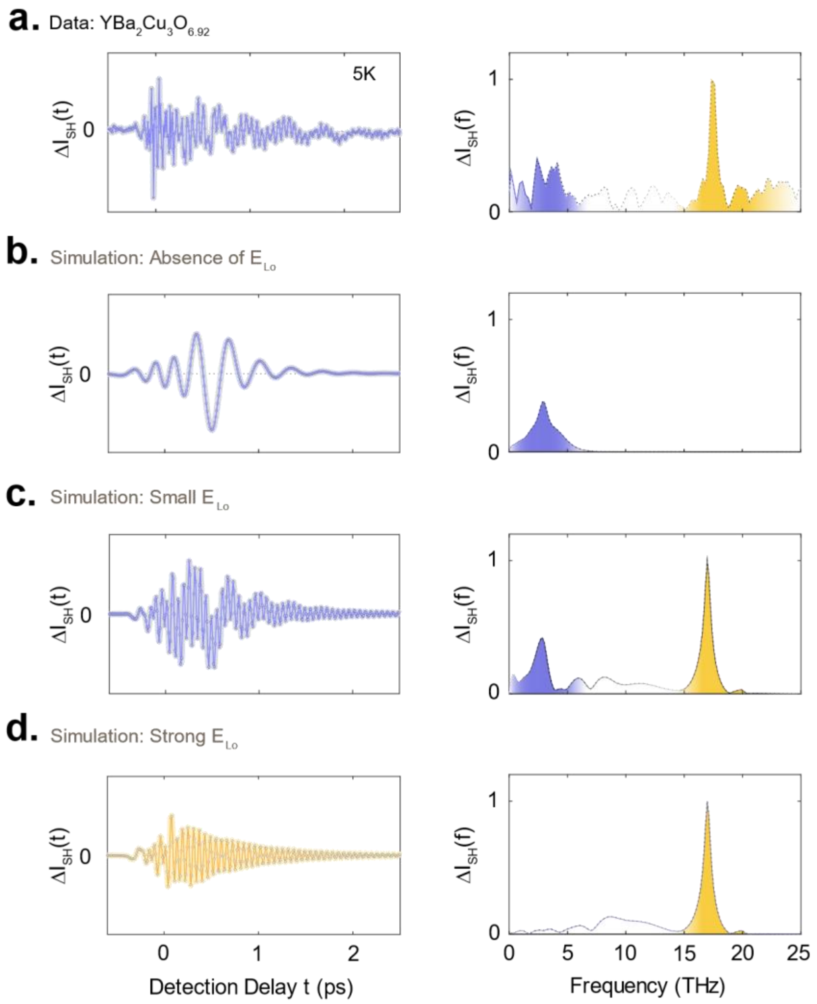

Supplementary Figure 1: Comparison between data and simulation for SHG measurement in $YBa_2Cu_3O_{6.92}$. (a) Left panel: oscillatory component of the mid-IR pump induced changes in time-resolved SHG intensity as a function of detection time delay t for $YBa_2Cu_3O_{6.92}$ at base temperature 5 K below T_c (T_c = 91K), at an excitation fluence of 29 mJ/cm^2. Right panel: corresponding Fourier spectrum of the data shown in the left panel, with two peaks at the resonantly excited phonon modes at 17 THz and 20 THz (shaded in yellow) and one smaller peak at the difference frequency of the two phonon modes ~ 3 THz (shaded in blue). **(b)** Left panel: simulated oscillatory component of the changes in second harmonic intensity for the lattice potential defined in Equation (2.1), in the homodyne detection limit. Right panel: corresponding Fourier spectrum with the same colour coding as in (a). Only the beating between the two phonon modes at 3 THz contributes to this spectrum. **(c)** Left panel: same as left panel in (b), now in the intermediate detection limit with a small, but nonzero local oscillator. Right panel: Same as right panel in (b). Here, the two phonon modes also appear at their fundamental frequencies. **(d)** Left panel: same as left panels in (b) and (c), now in the heterodyne detection limit with a strong local oscillator. Right panel: Same as right panels in (b) and (c). Here, the heterodyne contribution of the two phonon modes dominates the SHG signal.

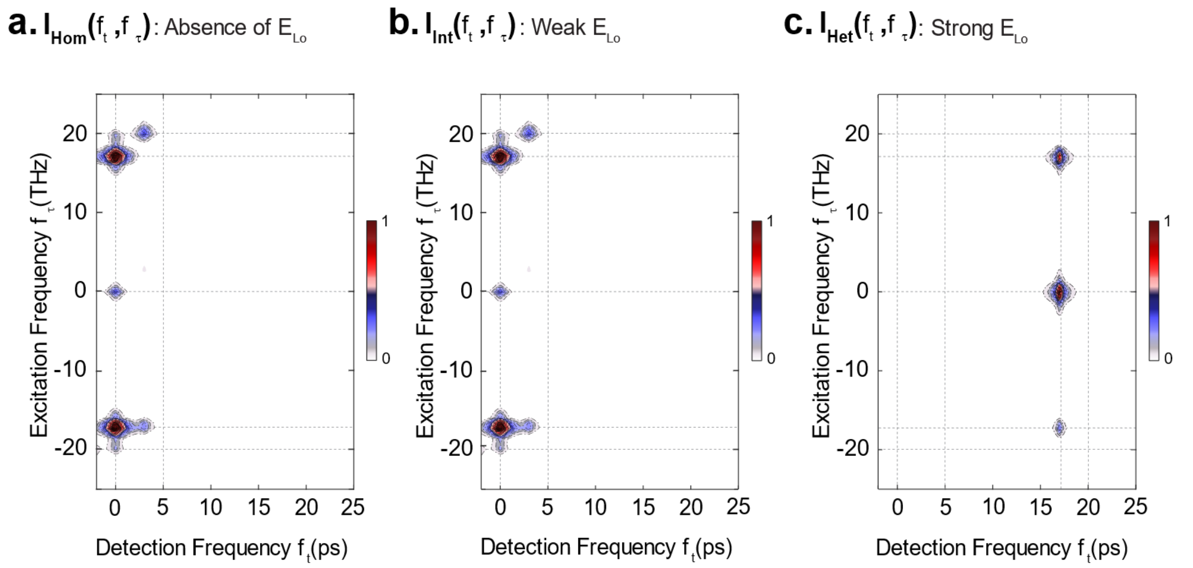

Supplementary Figure 2: Simulated nonlinear 2D spectrum of the phonon-phonon dynamics. (a) Two-dimensional Fourier spectrum simulated using the lattice potential defined in Eq. (2.1) for two-pulse excitation in the limit of homodyne. There are nine peaks in the spectrum with corresponding frequency coordinates (0;17), (0;20), (3;20), (0;0), (0;-17), (0;-20) and (3;-17) all in units of THz. **(b)** Same as in (a) for a non-zero, but small local oscillator. The peaks coordinates are the same as in (a). **(c)** Same as in (a) & (b) in the limit of heterodyne detection with strong local oscillator. The peak coordinates found in this spectrum are (17;0), (17:17) and (17:-17), all in units of THz. Due to the strong local oscillator, the nonlinear SHG intensity at the resonance frequency of the phonon modes is recovered. Panels (a), (b) and (c) were normalized according to their own maximum values.

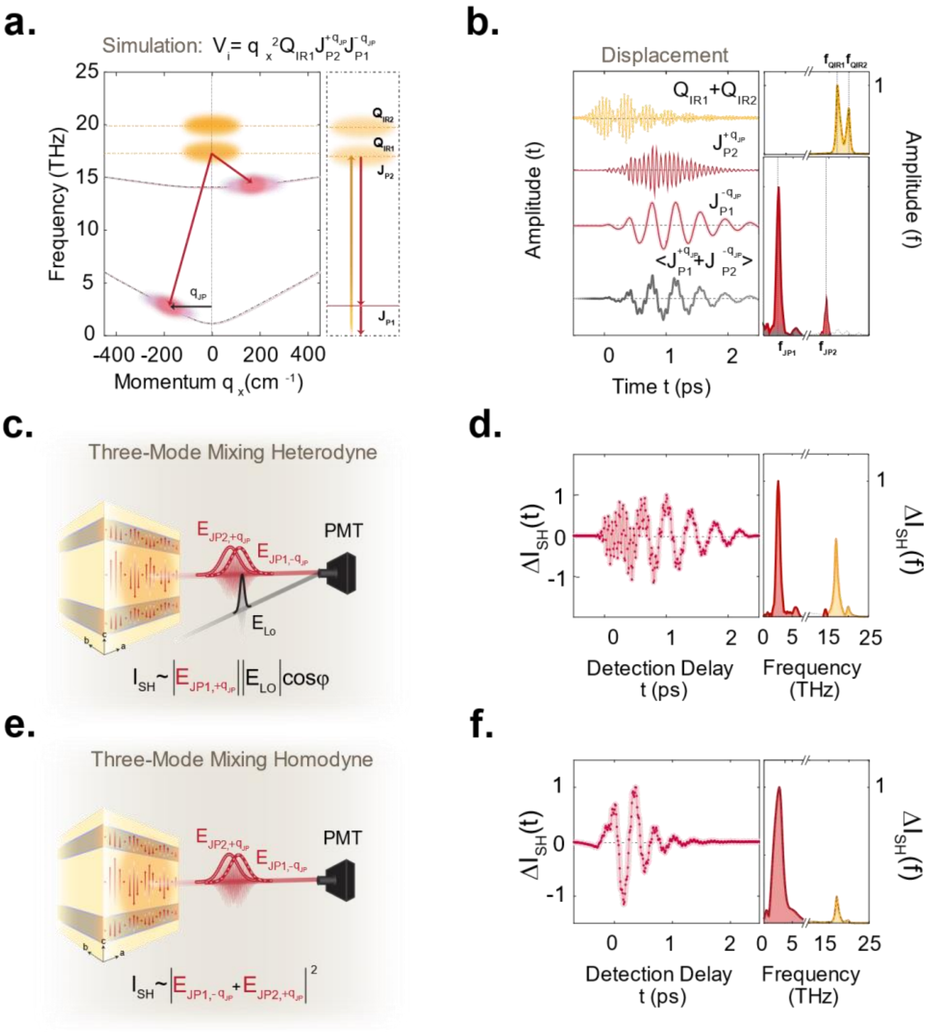

Supplementary Figure 3: Summary of the heterodyne and homodyne detections for three-wave mixing model. (a) Left panel: dispersion curves of the two apical oxygen phonon modes (Q_{IR1} at 17 THz and Q_{IR2} at 20 THz) and of the inter-bilayer (J_{P1}) and intra-bilayer (J_{P2}) Josephson plasma polaritons along the in-plane momentum q_x are shown as yellow and red dashed lines, respectively. The mid-IR pump excites both apical oxygen phonon modes, which parametrically amplify a pair of inter-bilayer and intra-bilayer Josephson plasma polaritons at finite momentum q_{JP} (black arrow) such that $\omega_{IR1} = \omega_{JP1}(-q_{JP}) + \omega_{JP2}(q_{JP})$. The right panel depicts the energy level diagram corresponding to this model. **(b)** Time-dependent displacement of phonon modes, the two Josephson plasma polaritons and their average value following the photoexcitation, simulated using the three-mode mixing model (left panel) with their respective Fourier spectrum (right panel). The yellow shading indicates the two driven apical oxygen phonons while the two red shading is attributed to Josephson plasma polaritons. **(c)** Second harmonic generation heterodyne detection configuration in the presence of local oscillator field E_{LO}. The hyper-Raman fields generated in the sample interfere with E_{LO} at the detector. **(d)** Simulated oscillatory component of the changes in second harmonic intensity of the Josephson plasmons supercurrents and the driven phonons in the heterodyne detection limit. Left panel: the time-delay dependent second

harmonic intensity in the heterodyned detection limit following apical oxygen phonon excitation, simulated using the three-mode mixing model detailed in Supplementary Note 3.A., and the corresponding Fourier spectrum using the same color shading as in (b). **(e)** Second harmonic generation homodyne detection configuration in the absence (negligible amount) of local oscillator field E_{LO}. In this limit, the hyper-Raman fields emitted from the sample are measured as an intensity by the detector. **(f)** Same as in (d) but showing the oscillatory component in the homodyne detection limit (small amplitude of local oscillator) using the same color shading as in (b) and (c).

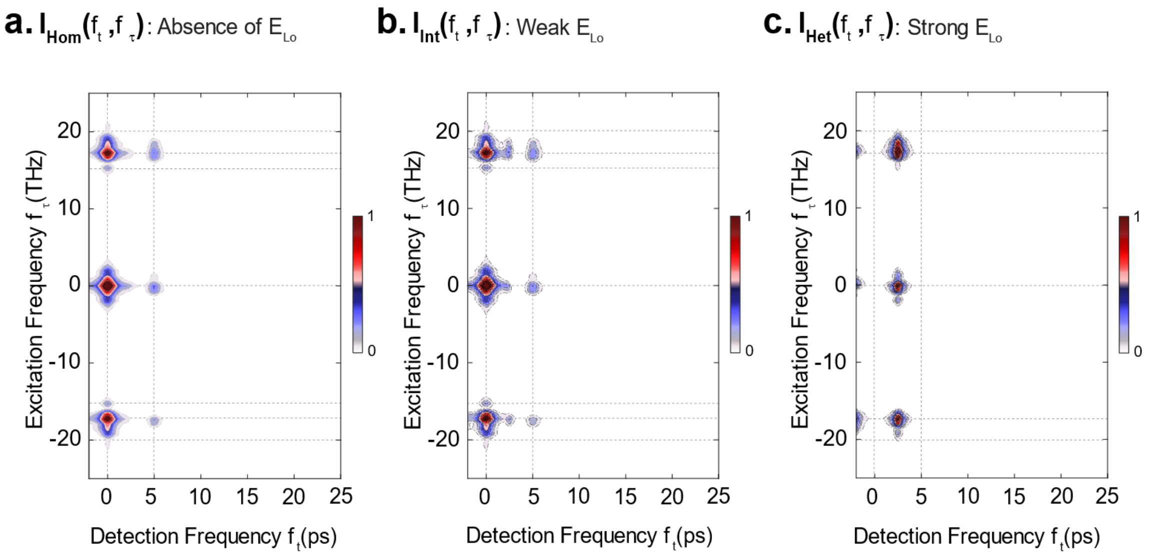

Supplementary Figure 4: Simulated nonlinear 2D spectrum of phonon-plasmon dynamics via the three-mode mixing model. (a) Two-dimensional Fourier spectrum simulated using the lattice potential defined in Eq. (3.1) for two-pulse excitation in the limit of homodyne detection. In this 2D spectrum, nine peaks can be found with frequency coordinates (f_t;f_τ); (0;14.5), (0;17), (0;20), (0;-14.5), (0;-17), (0;-20), (0;0), (5;0), (5;17), and (5;-17), all in units of THz. **(b)** Same as in (a) for a non-zero, but small local oscillator. In this 2D spectrum, there are 12 peaks, nine of which are the same as in (a) with extra peaks at frequency coordinates; (2.5;0), (2.5;17) and (2.5; -17), all in units of THz. **(c)** Same as in (a) & (b) in the limit of heterodyne detection with a strong local oscillator. In this 2D spectrum, there are only six peaks with the strong amplitude, at frequency coordinates; (2.5;0), (2.5;17) and (2.5;-17), all in units of THz. Panels (a), (b) and (c) were normalized according to their own maximum values.

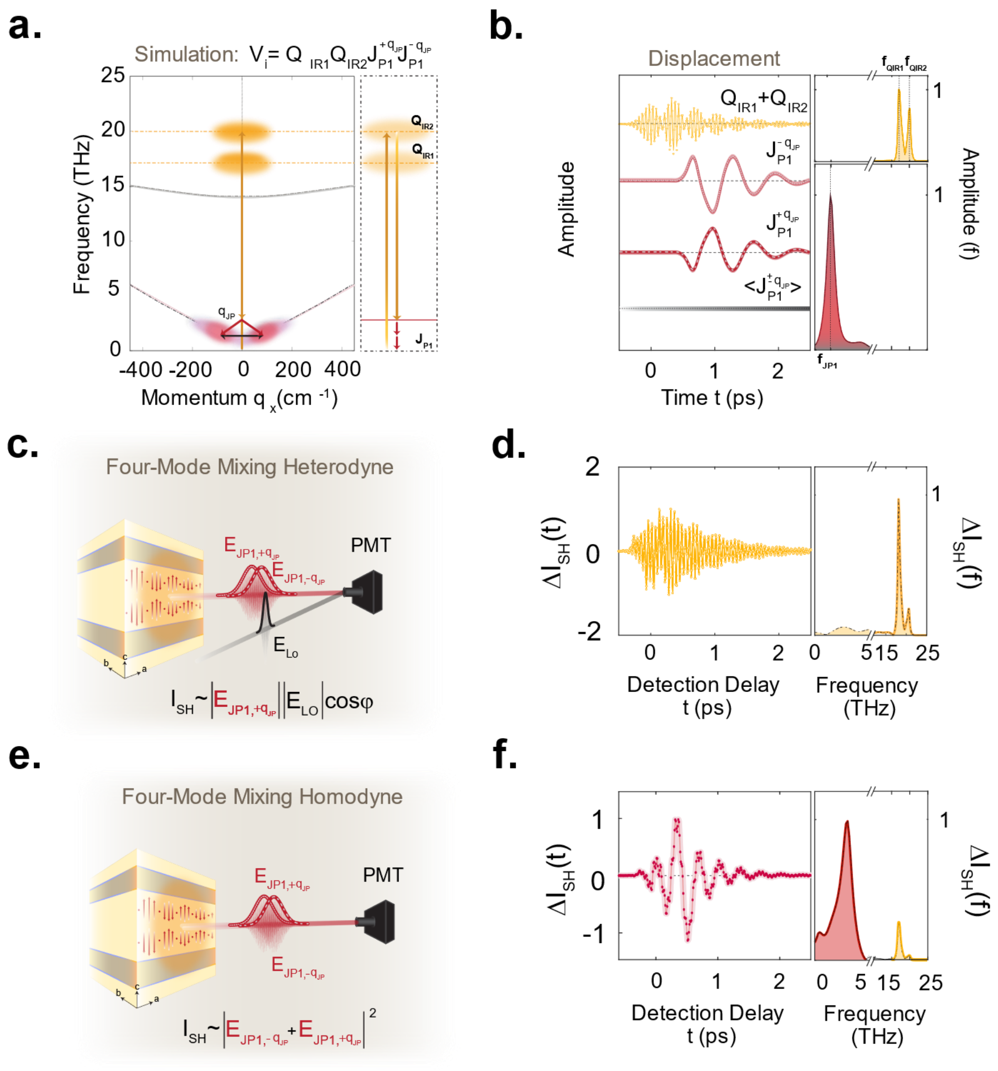

Supplementary Figure 5: Summary of the heterodyne and homodyne detections for four-wave mixing model. (a) Left panel: same dispersion relations as in Supplementary Figure 4(a) now for the four-mode mixing model explained in the text. The mid-IR pump again excites the two apical oxygen phonon modes. Now, they parametrically amplify a pair of inter-bilayer Josephson plasmon polaritons (J_{P1}) at finite momentum $\pm q_{JP}$ (illustrated by the black arrow) such that $\omega_{IR2} - \omega_{IR1} = 2\omega_{JP1}(\pm q_{JP})$ as explained in Supplementary Note 3.B. The right panel shows the energy level diagram describing the four-mode mixing model. **(b)** Time-dependent displacement of phonon modes (in yellow), the two Josephson plasma polaritons (in light dotted red and dark dashed red) and their average value following the photoexcitation (in grey), simulated using the four-mode mixing model (left panel) with their respective Fourier spectrum (right panel). **(c)** Heterodyne detection configuration of the second harmonic generation in the presence of local oscillator field at the detector E_{LO}. **(d)** Second harmonic generation measurement of the four-mode mixing dynamics. As the averaged response of Josephson plasmons supercurrents is zero, the simulated SH intensity in this limit has only contributions form the two driven phonon modes. **(e)** The homodyne detection configuration of the second harmonic generation in the absence (negligible amount of E_{LO}). Here, the generated fields in the sample are measured as an intensity. **(f)** Same as in (c) but showing the oscillatory component in the homodyne detection limit using the same color coding as in (b) and (c).

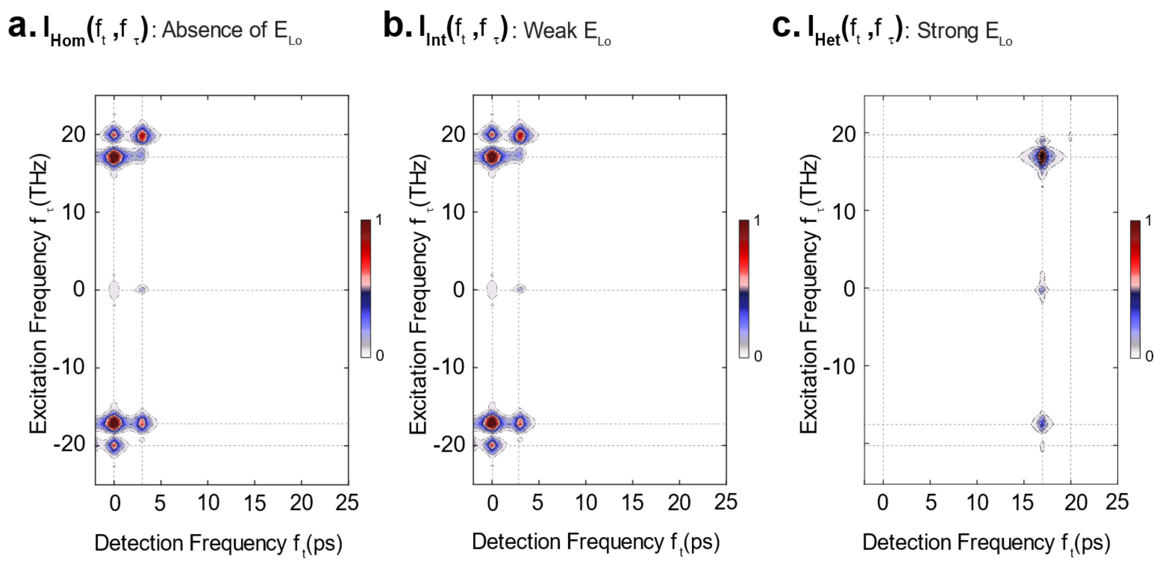

Supplementary Figure 6: Simulated nonlinear 2D spectrum of the phonon-plasmon dynamics via the four-mode mixing model. (a) Two-dimensional Fourier spectrum simulated using the lattice potential defined in Eq. (3.3) for two-pulse excitation in the limit of homodyne detection. According to this 2D spectrum, there are eight strong peaks at frequency coordinates ($f_t;f_\tau$); (0;17), (0;20), (3;20), (0;-17), (0;-20), (3;-17) and (0;0) all in units of THz. **(b)** Same as in (a) for a non-zero, but small local oscillator with the same peak pattern and frequency coordinates as in (a). **(c)** Same as in (a) & (b) in the limit of heterodyne detection with a strong local oscillator. Here, the number of peaks reduced to four peaks with frequency coordinates; (17;0), (17;17), (20;20) and (17; -17) all in units of THz. The heterodyne detected nonlinear SHG response for the plasmon averages to zero due to the fluctuating seed. Panels (a), (b) and (c) were normalized according to their own maximum values.

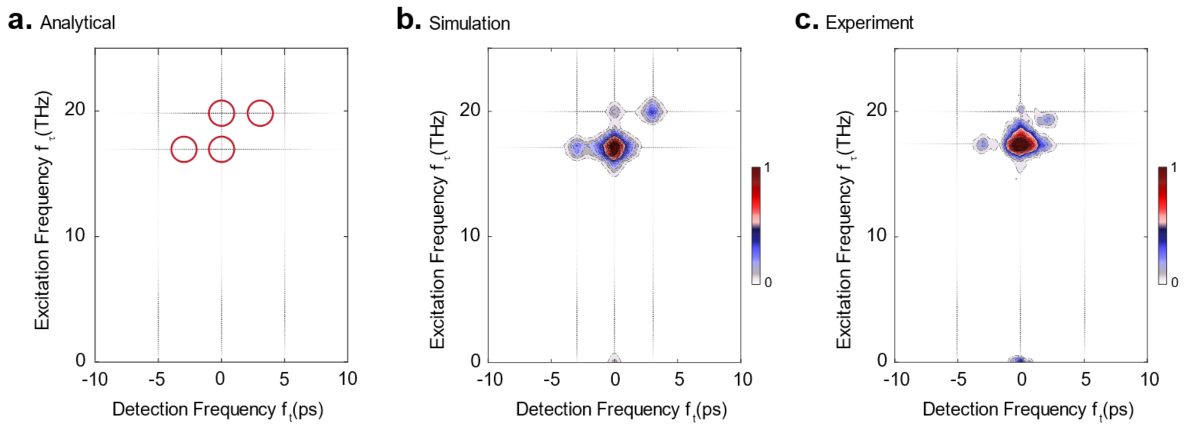

Supplementary Figure 7: Nonlinear 2D spectrum of the phonon dynamics predicted by the analytical approach. **(a)** Predicted peak pattern obtained from Eq. (5.18), using the two phonon mode coordinates' first order response and calculated in the homodyne SHG intensity detection limit. Within the experiment resolution, there are four peaks with the corresponding frequency coordinates (f_t;f_τ); (($\Omega_{IR2} - \Omega_{IR1}$); Ω_{IR2}), (($\Omega_{IR1} - \Omega_{IR2}$); Ω_{IR1}), (0; Ω_{IR2}), (0; Ω_{IR1}), all in units of THz. **(b)** Two-dimensional Fourier spectrum simulated using the lattice potential defined in Eq. (2.1) for two-pulse excitation (considering small excitation fields $E_{0A} = E_{0B}$ as in the analytical solution) in the limit of homodyne detection. **(c)** Two-dimensional Fourier spectrum measured in $YBa_2Cu_3O_{6.92}$ at base temperature 20 K below T_c shown in Fig. 6(c) of the main text. Panels (b) and (c) were normalized according to their own maximum values. The predicted, simulated and measured peak patterns are in good agreement.

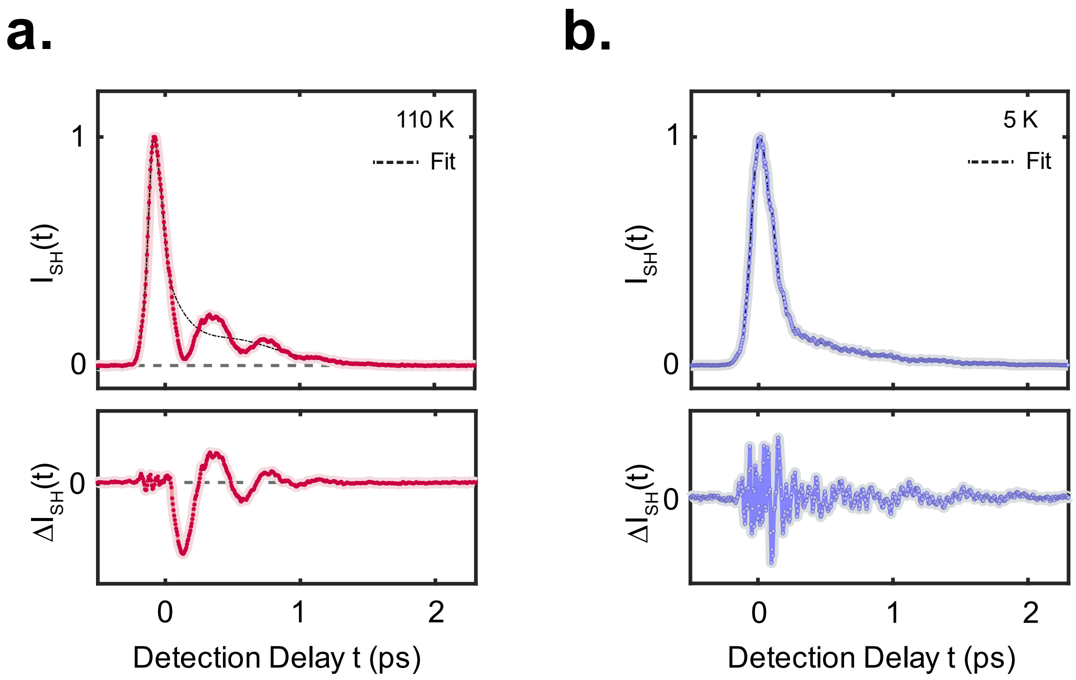

Supplementary Figure 8: Extracting mode oscillations from the tr-SHG intensity. **(a)** Upper plot: Normalized one-dimensional time-resolved SHG intensity measured in $YBa_2Cu_3O_{6.48}$ at base temperature 110K above T_c (red dotted curve) and a corresponding fit to the data, described in Eq. (6.1) to subtract the slowly varying background (dashed black curve). Lower plot: The residual of the fitted curve shown above and in the main text Fig. 1(e). **(b)** Upper Plot: same as the first panel in (a) for $YBa_2Cu_3O_{6.92}$ at base temperature 5K below T_c (blue dotted curve). Lower plot: same as the lower plot in (a), shown in the main text Fig. 6(a).

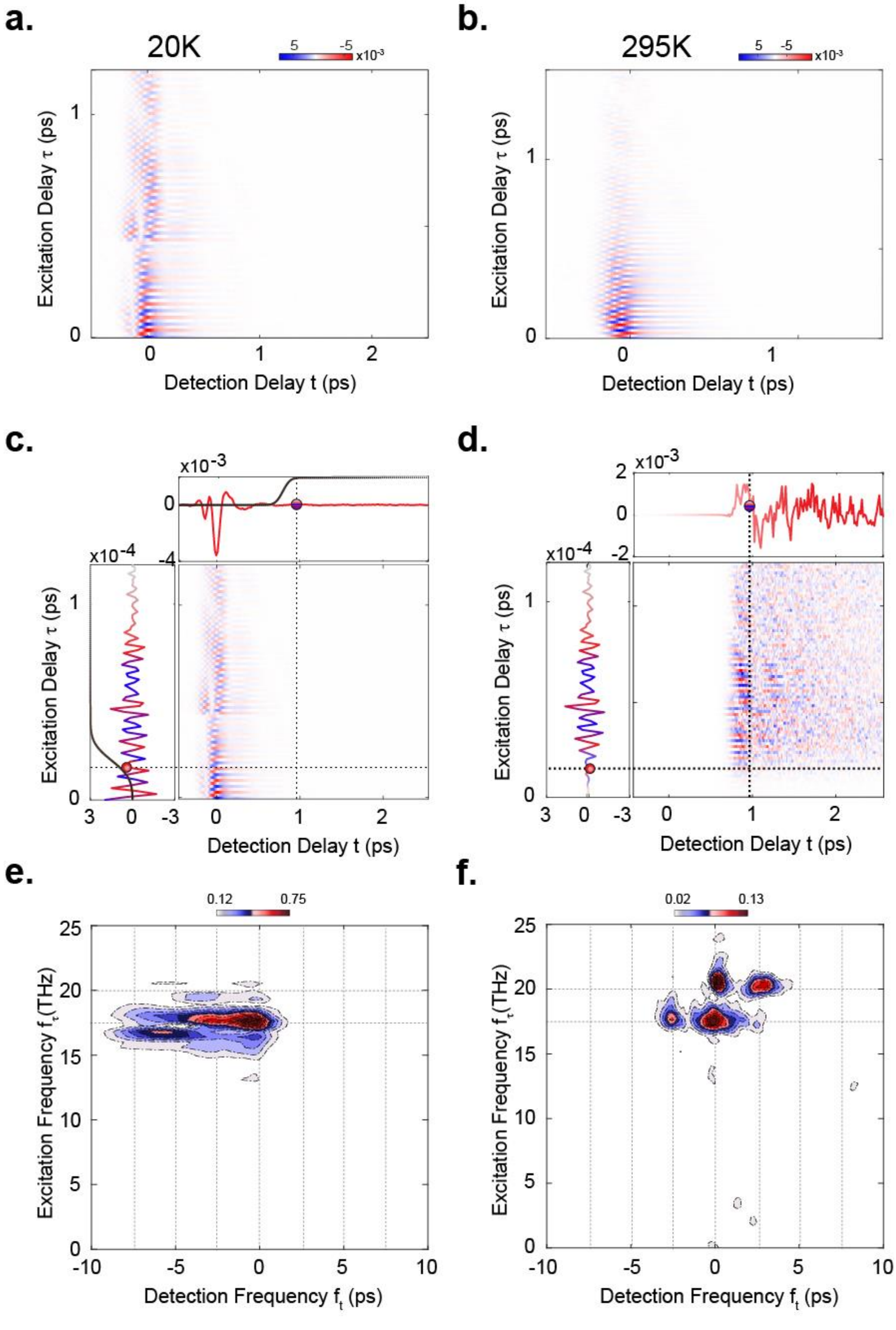

Supplementary Figure 9: Two-dimensional nonlinear spectroscopy of the SHG response in $YBa_2Cu_3O_{6.48}$ and the corresponding analysis. (a) uncropped nonlinear contributions to the time-resolved SHG intensity measured in $YBa_2Cu_3O_{6.48}$ at base temperature 20 K below T_c, shown in the main text Fig. 4(a). **(b)** Same as in (a) measured at base temperature 295 K above T_c, shown in the main text Fig. 4(b). **(c)** Central plot: same as in (a) with vertical (red-blue-shaded line) and horizontal (red-shaded line) slices of the data. Top plot: a horizontal slice of the data showing the nonlinear time-resolved SHG intensity as a function of detection delay (t) for a chosen

excitation delay (τ = 0.13 ps). Left plot: a vertical slice of the data showing the nonlinear time-resolved SHG intensity as a function of excitation delay (τ) for a chosen detection delay (t = 0.96 ps). The black curve in both top and left plots indicates the smooth error function (discussed in Supplementary Note 6) used to crop the data along detection and excitation delay, respectively. **(d)** Central plot: same data as in (a) after cropping along both time axis. Top plot: the same horizontal slice of the data shown in (a) top panel after cropping, magnified by a factor of 40. Left plot: same vertical slice of the data as in (a) left panel after cropping, magnified by a factor of 5. **(e)** Normalized two-dimensional Fourier transform of the data shown in (a) before cropping. **(f)** Same as in (c) for (b) after cropping, shown in the main text Fig. 4(c) and Fig. 6(a). For comparison, data in (d) are normalized to (c).

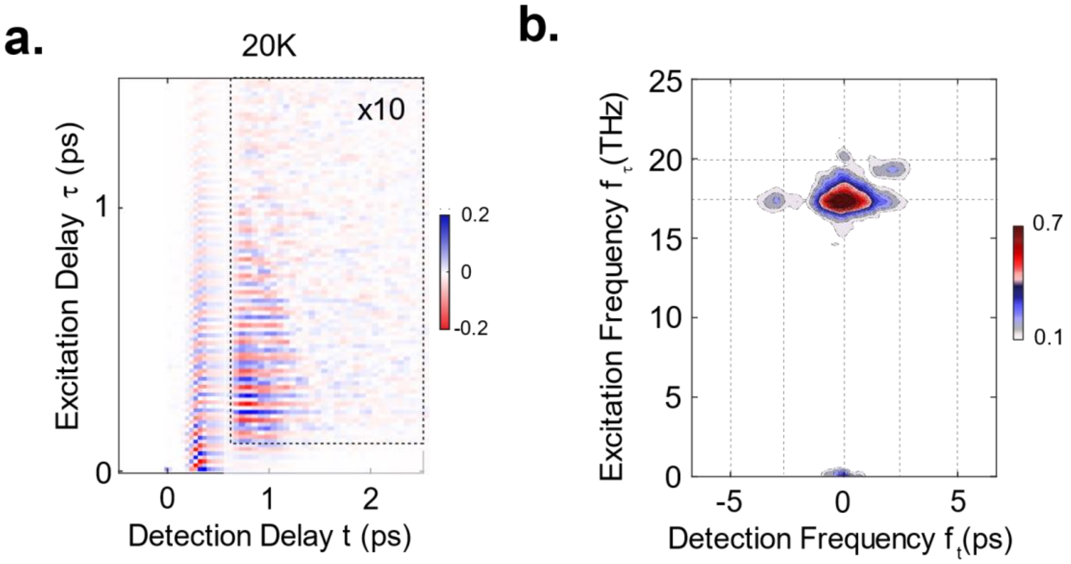

Supplementary Figure 10: Two-dimensional nonlinear spectroscopy of the SHG response in $YBa_2Cu_3O_{6.92}$. (a) Nonlinear contributions to the time-resolved SHG intensity with the excitation time delay τ changing along the vertical axis, measured at a base temperature of 20 K (below T_c). The data inside the black dashed box are multiplied by 10 for clarity. **(b)** Normalized two-dimensional Fourier spectrum of the data inside the black dashed box in panel (a), shown in the main text Fig 6(c).

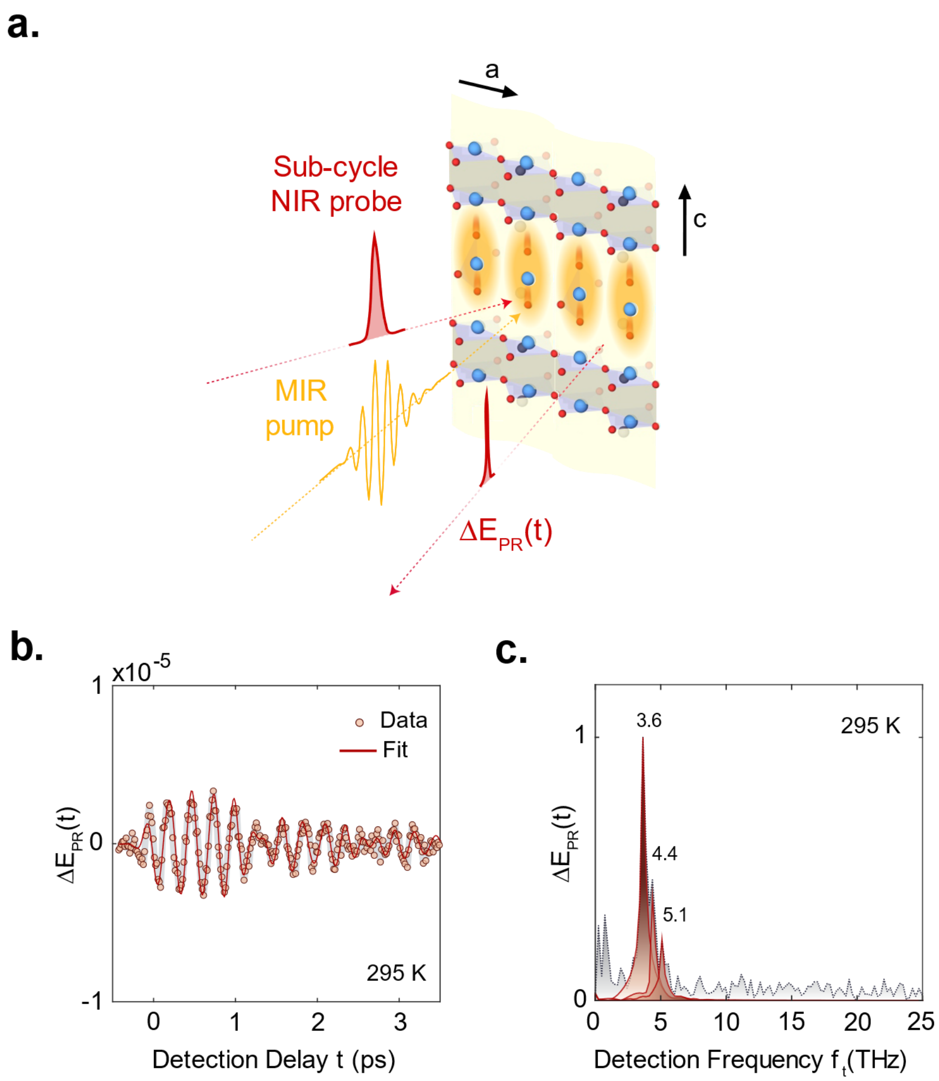

Supplementary Figure 11: Mid-IR pump, polarization rotation experiment in $YBa_2Cu_3O_{6.92}$. (a) Schematic of the mid-IR pump, polarization rotation (PR) experiment in $YBa_2Cu_3O_{6.92}$. The sample is excited by a mid-IR pump pulse (yellow) polarized along the crystal c-axis, resonantly exciting apical oxygen phonon modes as indicated by the yellow shading. The photo-induced changes of the polarization state of the reflected beam were sampled by a short near-infrared probe pulse at 800 nm wavelength (red). **(b)** The polarization rotation signal measured at base temperature of 295 K (above T_c) reveals coherent oscillations of Raman-active phonons. The red solid line is a fit to the data. **(c)** Corresponding Fourier spectra of the experimental data (shaded in grey) and the fit (shaded in brown) show three A_g Raman mode frequencies at 3.6 THz, 4.4 THz and 5.1 THz.

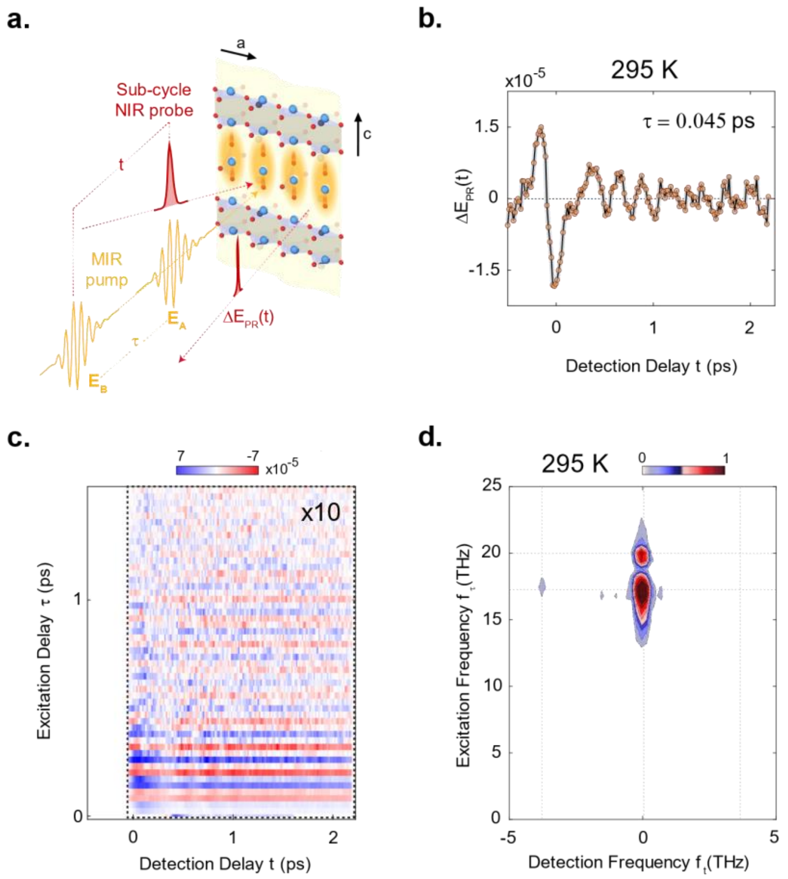

Supplementary Figure 12: Two-dimensional nonlinear spectroscopy of the polarization rotation response in $YBa_2Cu_3O_{6.92}$. (a) Schematic of the same experiment as shown in Supplementary Figure 9(a), now using two CEP stable mid-IR pump pulses E_A and E_B separated by the excitation delay τ with individual excitation fluences of 3 mJ/cm^2. **(b)** Nonlinear contribution to the time-resolved polarization rotation signal obtained via $\Delta E_{PR,NL} = \Delta E_{PR,AB} - \Delta E_{PR,A} - \Delta E_{PR,B}$, measured at base temperature of 295 K (above T_c), indicating the nonlinear excitation of the coherent A_g Raman mode oscillations. **(c)** Nonlinear contribution to the time-resolved polarization rotation signal (as described in (b)) with the excitation time delay τ changing along the vertical axis. The data inside the black dashed box are multiplied by 10 for clarity. **(d)** Normalized two-dimensional Fourier spectrum of the data inside the black dashed box in panel (c), representing three peaks at frequency coordinates (0,17), (0,20) and (-3.6,17) all in units of THz.

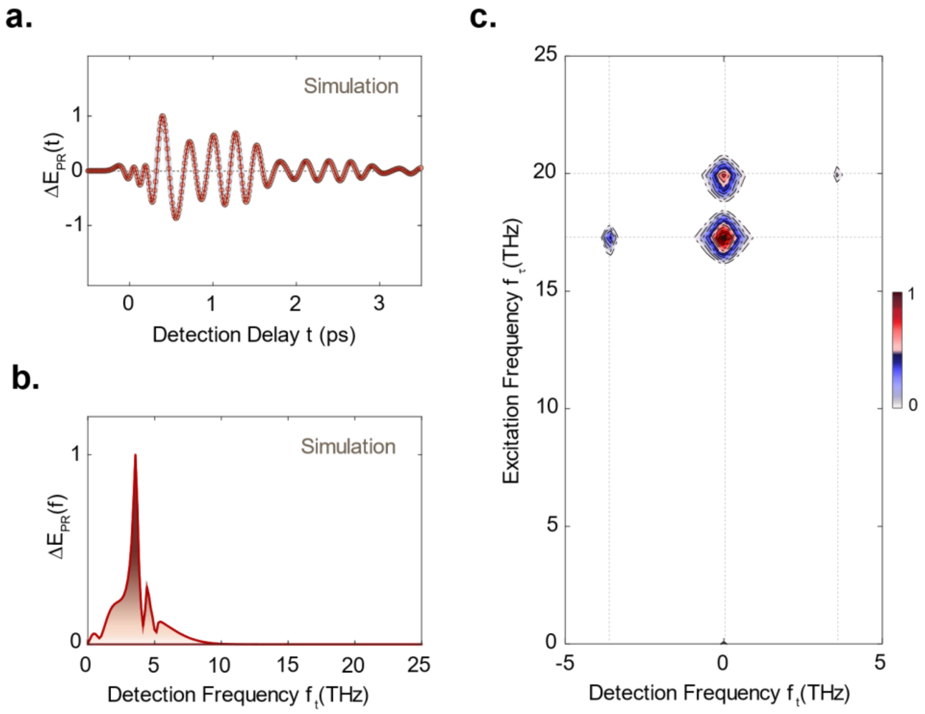

Supplementary Figure 13: Simulated pump-probe and two-dimensional nonlinear spectroscopy of phonon-phonon coupling in $YBa_2Cu_3O_{6.92}$. (a) The normalized polarization rotation response simulated using the lattice potential defined in Eq. (8.2) for phonon-phonon coupling in $YBa_2Cu_3O_{6.92}$, showing a good agreement with data shown in Supplementary Figure 12 (b) **(b)** Corresponding Fourier spectrum of (a) revealing three A_g Raman phonon modes at 3.6 THz, 4.4 THz and 5.1 THz, in agreement with Fig. S12 (c). **(c)** Simulated two-dimensional Fourier spectrum for the lattice potential defined in Eq. (6.2), showing three strong peaks at frequency coordinates (0,17), (0,20) and (-3.6,17) along with a much weaker on at (3.60, 20) all in units of THz, in agreement with the experimental data of Supplementary Figure 12 (d).

Supplementary References

1 Chang, Y. M., Xu, L. & Tom, H. W. K. Observation of Coherent Surface Optical Phonon Oscillations by Time-Resolved Surface Second-Harmonic Generation. *Physical Review Letters* **78**, 4649-4652, (1997).

2 Zhao, H. B. *et al.* Detection of coherent acoustic phonons by time-resolved second-harmonic generation. *Physical Review B* **83**, 212302, (2011).

3 Mankowsky, R., von Hoegen, A., Först, M. & Cavalleri, A. Ultrafast Reversal of the Ferroelectric Polarization. *Physical Review Letters* **118**, 197601, (2017).

4 Fiebig, M., Pavlov, V. V. & Pisarev, R. V. Second-harmonic generation as a tool for studying electronic and magnetic structures of crystals: review. *J. Opt. Soc. Am. B* **22**, 96-118, (2005).

5 Fechner, M. & Spaldin, N. A. Effects of intense optical phonon pumping on the structure and electronic properties of yttrium barium copper oxide. *Physical Review B* **94**, 134307, (2016).

6 Juraschek, D. M., Fechner, M. & Spaldin, N. A. Ultrafast Structure Switching through Nonlinear Phononics. *Physical Review Letters* **118**, 054101, (2017).

7 Homes, C. C., Timusk, T., Bonn, D. A., Liang, R. & Hardy, W. N. Optical properties along the c-axis of YBa2Cu3O6+x, for x = 0.50 → 0.95 evolution of the pseudogap. *Physica C: Superconductivity* **254**, 265-280, (1995).

8 von Hoegen, A. *et al.* Amplification of Superconducting Fluctuations in Driven $YBa_2Cu_3O_{6+x}$. *Physical Review X* **12**, 031008, (2022).

9 Michael, M. H. *et al.* Parametric resonance of Josephson plasma waves: A theory for optically amplified interlayer superconductivity in $YBa_2Cu_3O_{6+x}$. *Physical Review B* **102**, 174505, (2020).

10 Michael, M. H. *et al.* Generalized Fresnel-Floquet equations for driven quantum materials. *Physical Review B* **105**, 174301, (2022).

11 Michael, M. H. *et al.* Photonic time-crystalline behaviour mediated by phonon squeezing in Ta2NiSe5. *Nature Communications* **15**, 3638, (2024).

12 Först, M. *et al.* Nonlinear phononics as an ultrafast route to lattice control. *Nature Physics* **7**, 854-856, (2011).

13 Mankowsky, R. *et al.* Coherent modulation of the $YBa_2Cu_3O_{6+x}$ atomic structure by displacive stimulated ionic Raman scattering. *Physical Review B* **91**, 094308, (2015).

14 Disa, A. S., Nova, T. F. & Cavalleri, A. Engineering crystal structures with light. *Nature Physics* **17**, 1087-1092, (2021).
